# Internal exposure to heat-induced food contaminants in omnivores, vegans and strict raw food eaters: biomarkers of exposure to acrylamide (hemoglobin adducts, urinary mercapturic acids) and new insights on its endogenous formation

**DOI:** 10.1007/s00204-024-03798-z

**Published:** 2024-05-31

**Authors:** Bernhard H. Monien, Nick Bergau, Fabian Gauch, Cornelia Weikert, Klaus Abraham

**Affiliations:** Department of Food Safety, German Federal Institute for Risk Assessment (BfR), Max-Dohrn-Strasse 8-10, 10589 Berlin, Germany

**Keywords:** Human exposure, Acrylamide, Mercapturic acids, AAMA, GAMA, Hemoglobin adducts, Biomarker

## Abstract

The urinary mercapturic acids *N*-acetyl-*S*-(2-carbamoylethyl)-L-cysteine (AAMA) and *N*-acetyl-*S*-(2-carbamoyl-2-hydroxyethyl)-L-cysteine (GAMA) are short-term biomarkers of exposure from acrylamide and its metabolite glycidamide, respectively. The medium-term exposure to acrylamide and glycidamide is monitored by the adducts *N*-(2-carbamoylethyl)-Val (AA-Val) and *N*-(2-carbamoyl-2-hydroxyethyl)-Val (GA-Val) in hemoglobin (Hb), respectively. Three questions were addressed by application of these biomarkers in two diet studies including 36 omnivores, 36 vegans and 16 strict raw food eaters (abstaining from any warmed or heated food for at least four months): first, what is the internal acrylamide exposure following a vegan or a raw food diet in comparison to that in omnivores? Second, did the exposure change between 2017 and 2021? And third, what is the stability over time of AAMA/GAMA excretion compared to that of AA-Val/GA-Val levels in Hb between both time points? Median urinary AAMA excretion per day in non-smoking omnivores, vegans and raw food eaters were 62.4, 85.4 and 15.4 µg/day, respectively; the corresponding median AA-Val levels were 27.7, 39.7 and 13.3 pmol/g Hb, respectively. Median levels in strict raw food eaters were about 25% (AAMA excretion) and 48% (AA-Val) of those in omnivores. In comparison to 2017, AAMA and GAMA excretion levels were hardly altered in 2021, however, levels of AA-Val and GA-Val in 2021 slightly increased. There was a weak correlation between AAMA excretion levels determined four years apart (r_S_ = 0.30), and a moderate correlation between levels of AA-Val (r_S_ = 0.55) in this timeframe. Our data in strict raw food eaters confirm a significant endogenous formation to acrylamide in a size range, which is—based on the levels of AA-Val—distinctly higher than reported previously based on levels of urinary AAMA excretion. The relatively lower AAMA excretion in raw food eaters likely represents a lower extent of glutathione conjugation due to missing hepatic first-pass metabolism in case of endogenous formation of acrylamide, which leads to a higher systemic exposure.

**Supplementary Information:**

The online version contains supplementary material available at 10.1007/s00204-024-03798-z.

## Introduction

Acrylamide is a low-molecular weight, organic compound produced for different applications in chemical industry. Two decades ago, acrylamide was discovered to be formed in carbohydrate-rich food at elevated temperatures, e.g. through browning reactions that occur in the course of roasting, baking, or frying (Tareke et al. 2002). Particularly high levels were found in potato products, such as french fries and potato chips, in cereals, in bread (especially crispbread) and in coffee. Another main exposure source is tobacco smoke (EFSA 2015). In animal studies, acrylamide was shown to have neurotoxic, carcinogenic (NTP 2012), as well as genotoxic and mutagenic effects (Manjanatha et al. 2006; Mei et al. 2010). Prolonged or repeated occupational exposure led to neurotoxic effects in the peripheral nervous system (Pennisi et al. 2013). Although epidemiological studies have not consistently observed an increasing risk of common cancers in relation to dietary acrylamide intake (Filippini et al. 2022), there is concern about its potential carcinogenic effects in humans. The International Agency for Research on Cancer (IARC) classified acrylamide as *probably carcinogenic to humans* (group 2A) (IARC 1994).

Due to the genotoxic and carcinogenic properties of acrylamide and its metabolite glycidamide (EFSA 2022), the margin of exposure (MoE) concept was applied for the risk assessment of dietary exposure (EFSA 2005). MoE values calculated by the European Food Safety Authority (EFSA) were well below 1,000 for all age groups, and lower for children and infants compared to adults (EFSA 2015). It is of note, however, that the current debate on the mode of action and whether acrylamide should be considered as a genotoxic carcinogen may change the future risk assessment of human exposure (Guth et al. 2023).

In general, calculation of MoE values requires a reliable estimation of human exposure. Uncertainty of the latter is attributed to several factors, e.g. great variations in the content data, formation of acrylamide in domestic food preparations or the possibility that additional exposure via other routes, *i.e.* dermal (Kim et al. 2017; Sumner et al. 2003) and inhalative uptake (Manson et al. 2005; Urban et al. 2006), contributes significantly to human exposure. A potential circumvention of these problems is the use of biomarkers of internal exposure to heat-induced food contaminants, e.g. mercapturic acids (MAs) in urine samples (Mathias and B'Hymer 2016) or hemoglobin (Hb) adducts (Törnqvist et al. 1986) reflecting short-term (few days) or medium-term exposure (few months), respectively. The calculation of the consumer exposure after determining specific biomarkers in urine or blood samples (reverse dosimetry) (Clewell et al. 2008) can contribute to a more accurate exposure assessment by estimating the total internal dose, independently of the uptake route.

**Fig. 1:**
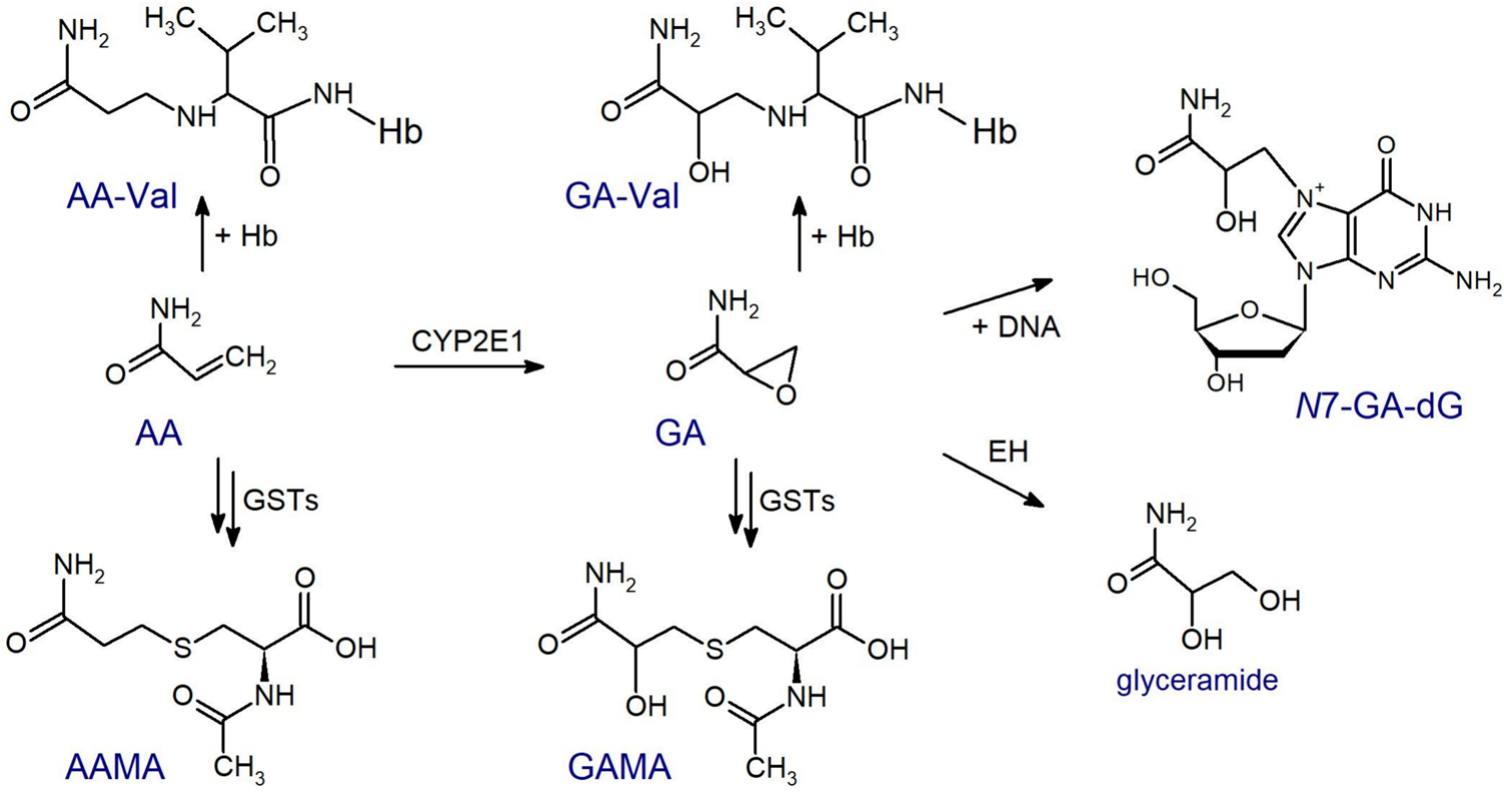
Acrylamide (AA) is metabolized to the genotoxic glycidamide (GA), most of which is metabolically inactivated by epoxide hydrolases (EH) to glyceramide. Acrylamide and glycidamide are also detoxified by glutathione S-transferases (GSTs). Degradation of the resulting conjugates leads to formation and urinary excretion of AAMA and GAMA, often used as biomarkers of short-term exposure. The medium-term exposure is monitored by the hemoglobin adducts AA-Val and GA-Val

The MAs formed from glutathione conjugates of acrylamide and its genotoxic metabolite glycidamide are *N*-acetyl-*S*-(2-carbamoylethyl)-L-cysteine (AAMA) and *N*-acetyl-*S*-(2-carbamoyl-2-hydroxyethyl)-L-cysteine (GAMA), respectively (Fig. 1). They are excreted in the urine and are typical short-term biomarkers of exposure (Boettcher and Angerer 2005; Goempel et al. 2017; Hays and Aylward 2008; Ruenz et al. 2016). The medium-term exposure to acrylamide and glycidamide can be monitored by analyses of the Hb adducts *N*-(2-carbamoylethyl)-Val (AA-Val) and *N*-(2-carbamoyl-2-hydroxyethyl)-Val (GA-Val), respectively (Fig. 1), after cleavage of the Val residues with a modified Edman degradation (Gauch et al. 2022; Paulsson et al. 2002; von Stedingk et al. 2010).

Both biomarkers have been used in multiple studies to uncover the influence of the intake of single foodstuffs (Bjellaas et al. 2007; Brantsæter et al. 2008; Hagmar et al. 2005; Outzen et al. 2011) and dietary or lifestyle habits (Fernandez et al. 2023; Goerke et al. 2019; Kotova et al. 2015; Vesper et al. 2007; Vikstrom et al. 2010) on the overall acrylamide exposure. The recent and ongoing shift from omnivore to vegetarian or vegan diets in western countries, believed to support an ecologically more sustainable lifestyle and to avoid suffering of farm animals, also raises questions on beneficial or adverse health effects. With respect to the latter, vegetarian and vegan diets may be accompanied by increased exposures to mycotoxins (Fleury et al. 2017; Penczynski et al. 2022) as well as microbial contaminants (Toth et al. 2021). With regard to heat-induced compounds, the daily urinary AAMA and GAMA excretion indicated on average a higher dietary exposure to acrylamide in vegans compared to omnivores (Goerke et al. 2019).

Another issue in the context of the internal exposure to acrylamide is its possible endogenous formation. First indications are from experimental investigations in rats showing urinary levels of AAMA and GAMA in untreated animals similar to those of ^14^C-AAMA and ^14^C-GAMA after application of a single oral dose of 0.1 µg ^14^C-acrylamide/kg bw (Watzek et al. 2012). In humans, significant urinary AAMA and GAMA excretions were observed following washout phases of three to five days with minimized dietary intake of acrylamide (Goempel et al. 2017; Ruenz et al. 2016).

**Fig. 2:**
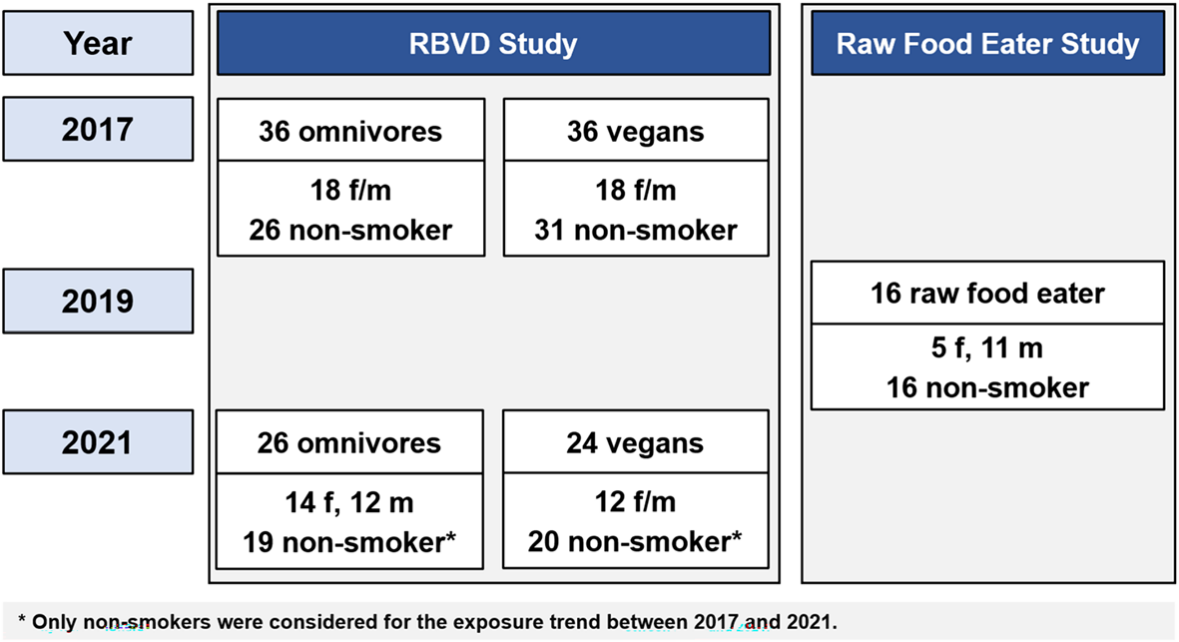
Blood and 24-h urine samples were collected from 88 adults in two different studies: In 2017, the Risks and Benefits of a Vegan Diet (RBVD) study included 36 vegans and 36 omnivores (Weikert et al. 2020), 50 of which were re-examined in 2021. The Raw Food Eater Study included 16 participants (Abraham et al. 2022)

This study had three goals: firstly, we analyzed the biomarkers mentioned in urine and blood of omnivores, vegans and strict raw food eaters, collected from adult participants in two studies (Fig. 2), to better understand the influence of the diet on internal exposure to acrylamide. The Risks and Benefits of a Vegan Diet (RBVD) study included 36 omnivores and 36 vegans (Weikert et al. 2020). In a separate study designed to investigate the internal exposure in the absence of dietary acrylamide (*i.e.* the contribution of endogenous formation of acrylamide as well as of other routes), sixteen strict raw food eaters were recruited who did not consume any food heated to higher temperatures than 42 °C (Abraham et al. 2022). Furthermore, 39 non-smoking participants of the RBVD study were re-examined four years later using the same methods. The two objectives of this follow-up investigation were to investigate the overall time trend of exposure to acrylamide, and to better understand the validity of the short- and medium-term biomarkers of acrylamide and their individual stability over time.

## Materials and methods

### Study populations and sample collection

Urine and blood samples were taken from 88 adults examined in two different studies (Fig. 2). The Risks and Benefits of a Vegan Diet (RBVD) study aimed to compare the nutrition and biomarker status of omnivores and vegans (n = 36 each, sex- and age-matched). Inclusion criteria were healthy subjects with an age between 30 and 60 years. The diet had to be followed for at least one year, and omnivores had to consume three servings of meat or two servings of meat and two servings of sausages a week. The 72 participants (36 males and 36 females) were examined in 2017 (Weikert et al. 2020). In 2021, 24 vegans and 26 omnivores followed the invitation to a follow-up investigation. For the evaluations of the time trend between 2017 and 2021, only the 39 non-smoking participants (19 omnivores, 20 vegans) were considered (Fig. 2).

The second study is a cross-sectional study especially designed for the investigation of internal exposure to heat-induced contaminants in people strictly avoiding consumption of foods containing heat-induced contaminants (raw food eater study). Inclusion criteria were healthy subjects (age between 20 and 65 years) following a strict raw food diet for at least four months, thereby avoiding any consumption of food warmed/heated above 42 °C (home-prepared and industrially produced/processed food). Exclusion criteria were smoking and any consumption of hot meals or beverages like coffee or tea. Sixteen strict raw food eaters (11 males and 5 females) were examined in 2019 (Abraham et al. 2022).

In both studies, information on nutritional habits and lifestyle factors were collected using questionnaires. Detailed dietary consumption was documented using three-day weighed food records directly before blood sampling and urine collection. All participants were instructed in detail how to collect urine over 24 h. The 24-h samples were collected in preservative-free plastic containers, starting on the day before the study visit. The samples were thoroughly mixed and weighed, and aliquots were stored at − 80 °C until analysis. Fasting blood samples were taken using EDTA tubes S-Monovette® (9 mL, Sarstedt, Numbrecht, Germany). They were centrifuged (2,500⋅g, 12 min) and the plasma was removed. The remaining erythrocytes were washed twice with 0.9% aqueous sodium chloride (2.5 mL) and lyzed by adding 2.5 mL of distilled water. The Hb content was determined with a HemoCue Hb 201 + analyzer (Radiometer, Willich, Germany), and the suspensions were stored at − 80 °C until analysis.

The RBVD study (no. EA4/121/16 and EA4/063/21) and the raw food eater study (No. EA4/040/19) were approved by the Ethics Committee of Charité University Medical Center Berlin. The raw food eater study (No. 00017436) and the RBVD follow-up study (No. 00025857) were registered in the German Clinical Trials Register (DRKS). The studies were performed in accordance with the ethical standards laid down in the 1964 Declaration of Helsinki and its later amendments (Abraham et al. 2022; Weikert et al. 2020). All participants got a detailed oral consultation about the rationales of the studies and gave informed consent in writing.

### Chemicals

Aqueous ammonia (25%), potassium hydrogen carbonate, tributylamine, acetic acid and hydrochloric acid (13 N), HPLC-grade acetonitrile, methanol and ethyl acetate, were obtained from Merck (Darmstadt, Germany). Formic acid (≥ 96%), *N*,*N*-dimethylformamide (DMF), dimethyl sulfoxide (DMSO), and fluorescein-5-isothiocyanate (FITC, isomer I, > 95%) were provided by Sigma (Steinheim, Germany) and water (UHPLC MS-Optigrade) was supplied by LGC Standards GmbH (Wesel, Germany). All reagents and solvents were of analytical grade.

The deuterated standard substances *N*-d_3_-acetyl-S-(2-carbamoylethyl)-L-cysteine (d_3_-AAMA) and *N*-d_3_-acetyl-S-(2-carbamoyl-2-hydroxyethyl)-L-cysteine (d_3_-GAMA) were purchased from Toronto Research Chemicals (Toronto, Canada). The isotope-labeled standards of the Hb adduct conjugates were custom synthesized. 3-(Fluorescein-5-yl)-1-(2-carbamoylethyl)-5-*d*_7_-isopropyl-2-thioxo-4-imidazolidinone (AA-*d*_7_-Val-FTH) was from Dr. Seidel at the Biochemical Institute for Environmental Carcinogens (Grosshansdorf, Germany). 3-(Fluorescein-5-yl)-1-(2-carbamoyl-2-hydroxyethyl)-5-*d*_7_-isopropyl-2-thioxo-4-imidazolidinone (GA-*d*_7_-Val-FTH) were prepared by the ASCA GmbH (Berlin, Germany). The dipeptide* N*-(2-carbamoyl-2-hydroxyethyl)-Val-Leu-anilide (GA-VL-An) was a generous gift from Dr. Schettgen (University of Aachen, Germany) and *N*-(2-carbamoylethyl)-Val-Leu-anilide (AA-VL-An) was provided by Bachem AG (Bubendorf, Switzerland).

### Preparation of standard solutions

The stock solutions of AA-*d*_7_-Val-FTH, GA-*d*_7_-Val-FTH and the respective dipeptides were prepared by accurate weighing of 1 mg dry material, which was dissolved in an adequate volume of DMSO to achieve solutions of 5 mmol/L. The working solutions of the isotope-labeled standards (50 nmol/L) and the dipeptides (100 nmol/L) were prepared by dilution in water/acetonitrile (1:1), aliquoted for further use and stored at − 80 °C.

Stock solutions of d_3_-AAMA (2 mg/L) and d_3_-GAMA (1 mg/L) were prepared after weighing of 1 mg dry material and dissolution in UHPLC-grade water. Aliquots of 400 µL d_3_-AAMA (2 mg/L) and 200 µL d_3_-GAMA (1 mg/L) were diluted in 100 mL UHPLC-grade water. Aliquots of the resulting working solution containing 8 µg/L d_3_-AAMA and 2 µg/L d_3_-GAMA were stored at − 80 °C.

### Edman degradation and solid-phase extraction (SPE)

The Hb adducts were analyzed following a modified Edman degradation using FITC for the cleavage of the N-terminal Val (Fig. S1) (Gauch et al. 2022; Rydberg et al. 2009). Briefly, aliquots of the erythrocyte samples (~ 35 mg Hb) were alkalized with 15 µL of 1 M aqueous potassium hydrogen carbonate. Ten µL of isotope-labeled standard solutions containing AA-*d*_7_-Val-FTH and GA-*d*_7_-Val-FTH (50 nmol/L), and 5 mg FITC dissolved in 30 μL DMF were added. Samples were incubated for 18 h at 37 °C. After adding 1.6 mL acetonitrile the samples were centrifuged (18,000**⋅**g, 10 min) and the pH of the supernatant was adjusted with 25 µL of 1 M aqueous ammonium hydroxide. The samples were transferred to Oasis MAX cartridges (60 mg; Waters, Eschborn, Germany), preconditioned with 2 mL acetonitrile and 2 mL water. After washing with acetonitrile, water and 1% aqueous formic acid (2 mL each), the FTH-conjugates of Val adducts were eluted with 3 mL acetonitrile/water (9:1) acidified with 1% formic acid. The extract sample was evaporated to dryness and reconstituted in 50 µL acetonitrile/water (1:1) containing 1% formic acid.

### UPLC–MS/MS analytical quantification of AA-Val-FTH and GA-Val-FTH

The FTH analytes were chromatographically separated using an Acquity UPLC system (Waters) and an Acquity HSS T3 column (1.8 µm, 2.1 mm × 150 mm, Waters). Aliquots of the samples (10 µL) were injected and eluted with water + 0.1% formic acid (eluent A) and acetonitrile + 0.1% formic acid (eluent B) applying a two-step gradient at a flow rate of 0.4 mL/min: 0—1 min (10% eluent B), 1–15 min (10–50% eluent B), 15–21 min (50–70% eluent B), 21–22.5 min (90% eluent B) and 22.5–24 min (10% eluent B). A triple quadrupole-hybrid ion trap mass spectrometer QTrap6500 (Sciex, Darmstadt, Germany) equipped with an electrospray ionization source operated in the positive mode was used for the detection of analytes and isotope-labeled reference substances by multiple reaction monitoring (MRM). The fragmentation transitions and the respective parameters (declustering potential, entrance potential, collision energies and cell exit potentials) are summarized in Table S1 of the Supplemental Information. Further mass spectrometric parameters were set to the following values: curtain gas, 20 psi; ion source temperature, 450 °C; ion spray voltage, 5500 V; ion source gas 1, 60 psi; ion source gas 2, 50 psi; collision-activated dissociation gas set to *medium*. The data was recorded and analyzed with Analyst 1.7.1 Software (Sciex).

The adduct levels were calculated as follows:

$${amount}_{adduct}\left[\frac{pmol}{g Hb}\right]=\frac{\frac{{A}_{analyte}}{{A}_{IS}} * {amount}_{IS} \left[pmol\right] }{{amount}_{Hb} \left[g\right]}$$

with A_analyte_ and A_IS_ as the peak areas of the quantifier signals of the analyte and of the internal standard, respectively, and with amount_IS_ and amount_Hb_ as the quantities of the internal standard and of Hb applied in the Edman degradation, respectively. The accuracy of quantification of AA-Val and GA-Val was improved using ten control samples of an erythrocyte pool (incorporated in each sample set). Five of these samples were spiked with 10 µL of the AA-VL-An and GA-VL-An solutions (100 nmol/L) and otherwise worked up as described. The analyses of these samples allowed determining the efficiency of the FITC-mediated Edman degradation, which were used to correct the levels of the two adducts determined in the human samples (Abraham et al. 2019; Monien et al. 2020).

### Ion-pair LC–MS/MS for the quantification of AAMA and GAMA

Urine samples were thawed and vortexed. Aliquots of 20 µL were diluted with 80 µL water and mixed with 100 µL of the internal standard solution (8 µg/L d^3^-AAMA; 2 µg/L d^3^-GAMA). The LC–MS/MS system consisted of an HPLC 1100 (Agilent, Waldbronn, Germany) connected to a mass spectrometer QTrap6500 (Sciex) via an electrospray source operated in the negative mode. The analytes were separated by ion-pair reversed-phase chromatography using a Nucleoshell RP 18plus (2.0 mm × 150 mm, 2.7 µm; Macherey–Nagel, Düren, Germany) and 10 mM tributylamine and 10 mM acetic acid in water (eluent A) and acetonitrile (eluent B) as eluents. The gradient was as follows: 0–1 min (2% eluent B), 1–12 min (2–75% eluent B), 12–19 min (75–100% eluent B), 19–21 min (100% eluent B), 21–21.1 min (100–2% eluent B), 21.1–24 min (2% eluent B), and the flow rate was 0.5 mL/min. The temperature of the column oven was set to 40 °C and the sample injection volume was 5 µL. Parameters for the detection of AAMA and GAMA and for the quantification with the respective isotope-labeled standards d_3_-AAMA and d_3_-GAMA (declustering potential, entrance potential, collision energies and cell exit potentials) are summarized in Table S2 of the Supplemental Information. The operating parameters of the QTrap6500 were: curtain gas, 40 psi; ion source temperature, 450 °C; ion spray voltage, 4500 V; ion source gas 1, 60 psi; ion source gas 2, 50 psi; collision-activated dissociation gas set to *medium*. Data acquisition and processing were carried out using Analyst 1.7.1 software (Sciex). The quality of quantification was confirmed by co-analyzing of an artificial urine sample with specified concentrations of AAMA and GAMA received from the German External Quality Assessment Scheme (G-EQUAS, Prof. Dr. Hans Drexler and Prof. Dr. Thomas Göen, Friedrich-Alexander University, Erlangen, Germany) in each batch.

### Statistical analysis

Biomarker level are reported as median values with the inter-quartile range (IQR) in brackets. Statistical standard procedures as described in the text were conducted using SigmaPlot 14.0 (Systat Software, Inc., Erkrath, Germany) or IBM SPSS Statistics (Version 26.0, Armonk, NY, USA). Differences with p values < 0.05 were considered statistically significant.

## Results

### Study populations

The 36 vegans and 36 omnivores of the RBVD study (18 women each) were aged between 30 and 57 years, with a mean age of 38.5 and 37.5 years in omnivores and vegans in 2017, respectively. The vegan participants had followed their diet between 1.6 years and 20.2 years (median 4.8 years). Further details are described elsewhere (Weikert et al. 2020). Smokers were defined by self-reporting (n = 13). For this evaluation, two users of E-cigarettes were also considered as smokers, leading to total number of 15 smokers (10 omnivores, 5 vegans).

Sixteen strict raw food eaters (5 women, 11 men) were recruited for the study. They had mean age of 44.6 years (range 23 to 63 years). On average, they followed their diet for 11.6 years (range 4 months to 29 years); nine participants (56%, one woman and 8 men) followed the diet for 10 years or longer. All participants were found to be well informed, for example, about processes during food production or which foods may have been heated before offered for sale. They were very convinced of their diet, recognizable by the usually long duration of adherence or by the daily burden they took on: to procure the right food (e.g., growing sprouts, shopping in specialist stores or via the Internet) or to do without many foods and most restaurant visits. The main nutritional basis of nearly all participants was the consumption of fresh fruit and raw vegetables; furthermore, 8 participants stated nuts as an important food. The participants can be classified as raw vegan (n = 4), as vegetarian (n = 4; they occasionally also consumed raw milk cheese or eggs), and omnivores (n = 8; they also ate raw meat and/or raw fish). Nonetheless, the number of eatable food groups was restricted in comparison to vegans and omnivores, as it was obvious from the 3-day weighed food records (only 23 of 57 food groups have been consumed by at least one of the participants). On the other hand, single food groups often have been consumed in large amounts, exceeding 1 kg per day in 9 participants (fresh fruit, raw vegetables, raw milk, or meat). Further details including description of the individual nutrition are described elsewhere (Abraham et al. 2022).

One surprising result in the group of raw food eaters was a urinary AAMA excretion of 601 µg/day in one of the male participants, much higher than the next highest value of 53.7 µg/day. The corresponding values of GAMA excretion were 26.6 and 6.50 µg/day, respectively. The results of AA-Val and GA-Val were unremarkable. After receiving these results, the participant was contacted and agreed to collect another 24-h urine. Its analysis showed a much lower urinary excretion of 8.33 µg AAMA and 0.46 µg GAMA per day. Evaluation of the initial 3-day food records revealed a high consumption of dried “Medjool” dates (506 g, 710 g and 1174 g on the days − 3, − 2 and − 1 before the study visit, respectively). Subsequently, the same product of “Medjool” dates was bought at the BfR, and the medical head of the unit (K.A.) consumed 284 g of these dates on an empty stomach in the morning. A distinct increase of urinary AAMA excretion was observed, from 5.7 µg in the 2 h sample before consumption to a maximum of 24.5 µg in the sample collected 8 to 10 h after consumption. After that, these dates were analysed for acrylamide at the Federal State Laboratory in Kassel,^1^ Germany, and revealed a content of 307 µg/kg. Of ten other dates products from the German market, the acrylamide content was quantifiable in two samples (55 and 26 µg/kg), the others were below the limit of detection. Even if the lot of the product analysed was not the same as that initially eaten by the participant the days before the study visit, the consumption of the dates very likely is the cause of the high urinary excretion initially observed. As the corresponding AA-Val and GA-Vals levels were not elevated, the consumption of dates with high acrylamide content in this participant was rated as exception. Therefore, his initial values of urinary AAMA and GAMA excretion were replaced by those of the second urine sample.

### Urinary AAMA and GAMA excretion in omnivores, vegans and raw food eaters

**Fig. 3:**
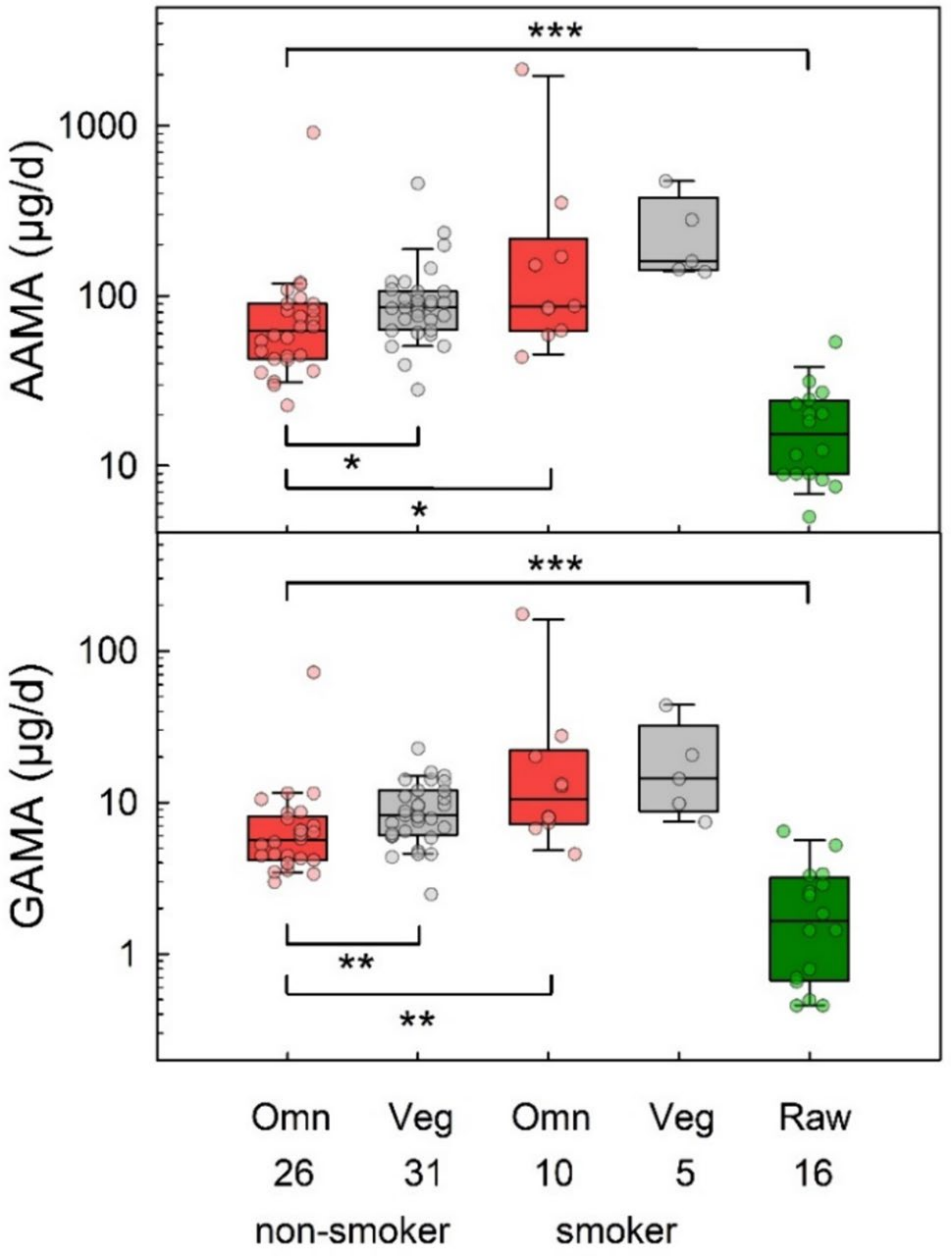
Daily excretion of AAMA (upper panel) and GAMA (lower panel) in non-smoking omnivore (red boxes, *n* = 26) and vegan (grey boxes, *n* = 31) study participants (RBVD study 2017), in the groups of smoking omnivores (red boxes, *n* = 10) and vegans (grey boxes, *n* = 5), and in strict raw food eaters (green boxes, *n* = 16). Lines and boxes represent median values and the lower and upper quartiles, respectively, and the bars represent the 10th and 90th percentiles. The significance of excretion differences is noted with asterisks (*p ≤ 0.05, **p ≤ 0.01, ***p ≤ 0.001, Mann–Whitney rank-sum test)

Figure 3 shows the urinary excretion of AAMA and GAMA in non-smoking and smoking omnivore and vegan participants of the RBVD study (2017) in comparison to that observed in (non-smoking) raw food eaters. The median amounts of AAMA excretion of non-smoking omnivores, vegans and raw food eaters were 62.4, 85.4, and 15.4 µg/day, respectively (Table 1). The corresponding amounts of urinary GAMA excretion were 5.7, 8.2, and 1.7 µg/day, respectively. The differences of AAMA (p = 0.015) and GAMA excretion (p = 0.004) between both non-smoking omnivores and vegans were statistically significant. The amounts of AAMA and GAMA excreted per day in raw food eaters were significantly different (p ≤ 0.001) compared to those observed for non-smoking omnivores.

**Table 1:** Minima, 25% percentile, median, 75% percentile and maxima of AAMA and GAMA excretion in smoking and non-smoking omnivores and vegans as well as strict raw food eaters determined in 24-h urine samples

		*n*	Min (µg/d)	25% (µg/d)	Median (µg/d)	75% (µg/d)	Max (µg/d)
AAMA	Omnivore/non-smoker	26	22.8	42.6	62.4	90.1	916
	Vegan/non-smoker	31	28.2	63.0	85.4	106	460
	Omnivore/smoker	10	43.8	61.8	86.5	216	2150
	Vegan/smoker	5	139	141	160	377	474
	Raw food eater	16	5.0	9.0	15.4	24.2	53.7
GAMA	Omnivore/non-smoker	26	3.0	4.2	5.7	8.1	72.9
	Vegan/non-smoker	31	2.5	6.1	8.2	12	22.9
	Omnivore/smoker	10	4.6	7.3	10.5	22.2	175
	Vegan/smoker	5	7.5	8.7	14.4	32.4	44.1
	Raw food eater	16	0.5	0.7	1.7	3.2	6.5

In the groups of smokers, the median urinary AAMA excretion was 86.5 µg/day in omnivores and 160 µg/day in vegans (Table 1). The corresponding amounts of urinary GAMA excretion were 10.5 and 14.4 µg/day, respectively. The differences of AAMA/GAMA excretion observed for non-smokers and smokers were significant in omnivores (p = 0.033/0.003) and vegans (p = 0.003/0.044).

### AA-Val and GA-Val in blood samples of omnivores, vegans and raw food eaters

**Fig. 4:**
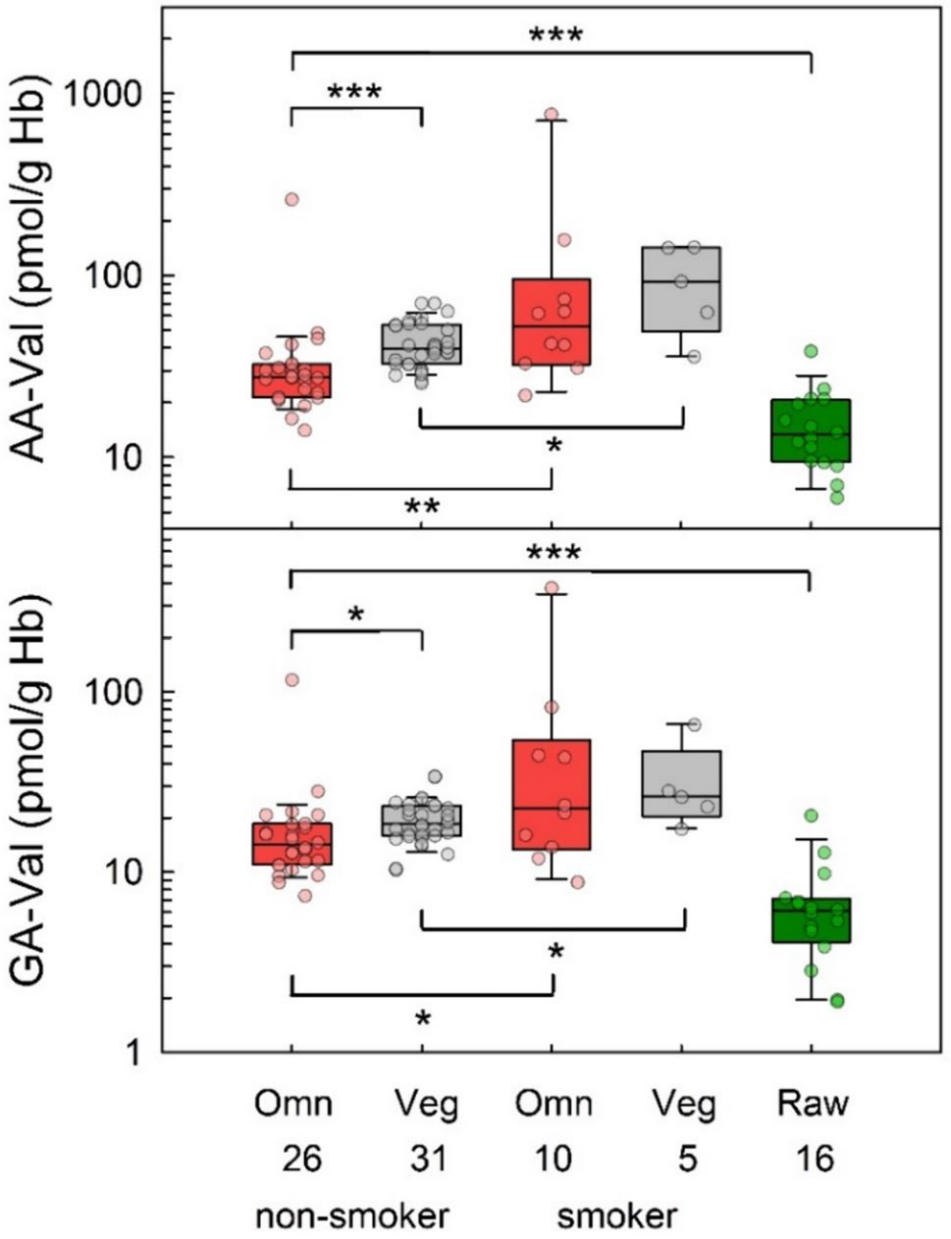
Levels of AA-Val (upper panel) and GA-Val (lower panel) in non-smoking omnivore (red boxes, *n* = 26) and vegan (grey boxes, *n* = 31) study participants (RBVD study 2017), in the groups of smoking omnivores (red boxes, *n* = 10) and vegans (grey boxes, *n* = 5), and in strict raw food eaters (green boxes, *n* = 16). Lines and boxes represent median values and the lower and upper quartiles, respectively, and the bars represent the 10th and 90th percentiles. The significance of adduct level differences is noted with asterisks (*p ≤ 0.05, **p ≤ 0.01, ***p ≤ 0.001, Mann–Whitney rank-sum test)

Figure 4 shows AA-Val and GA-Val levels in blood samples of non-smoking and smoking omnivores and vegans of the RBVD study (2017) and of the (non-smoking) participants of the raw food eater study. The median AA-level in blood samples of non-smoking omnivores, vegans and raw food eaters were 27.7, 39.7, and 13.3 pmol/g Hb, respectively (Table 2). The corresponding levels of GA-Val were 14.2, 18.5, and 6.1 pmol/g Hb, respectively. The AA-Val/GA-Val levels of non-smoking omnivores and vegans were statistically different (p < 0.001/0.006), and AA-Val/GA-Val levels of non-smoking omnivores and raw food eaters were statistically different (p < 0.001 each).

**Table 2:** Minima, 25% percentile, median, 75% percentile and maxima of AA-Val and GA-Val in Hb samples of in smoking and non-smoking omnivores and vegans as well as strict raw food eaters

		*n*	Min (pmol/g Hb)	25% (pmol/g Hb)	Median (pmol/g Hb)	75% (pmol/g Hb)	Max (pmol/g Hb)
AA-Val	Omnivore/non-smoker	26	14.1	21.4	27.7	32.5	262
	Vegan/non-smoker	31	25.6	32.7	39.7	53.6	70.5
	Omnivore/smoker	10	22.0	32.4	52.2	95.0	770
	Vegan/smoker	5	35.9	49.3	92.8	143	143
	Raw food eater	16	6.0	9.5	13.3	20.7	38.5
GA-Val	Omnivore/non-smoker	26	7.4	11.0	14.2	18.6	118
	Vegan/non-smoker	31	10.3	15.9	18.5	23.4	34.2
	Omnivore/smoker	10	8.8	13.4	22.6	54.3	378
	Vegan/smoker	5	17.4	20.3	26.3	47.2	66
	Raw food eater	16	1.9	4.1	6.1	7.2	20.7

In the group of smokers, the median AA-Val levels were 52.2 pmol/g Hb in omnivores and 92.8 pmol/g Hb in vegans. The corresponding GA-Val levels were 22.6 and 26.3 pmol/g Hb, respectively (Table 2). The differences of AA-Val/GA-Val excretion between non-smokers and smokers were significant in omnivores (p = 0.003/0.042) and vegans (p = 0.015/0.035).

### Ratios of AAMA/AA-Val and GAMA/GA-Val of omnivores, vegans and raw food eaters

**Fig. 5:**
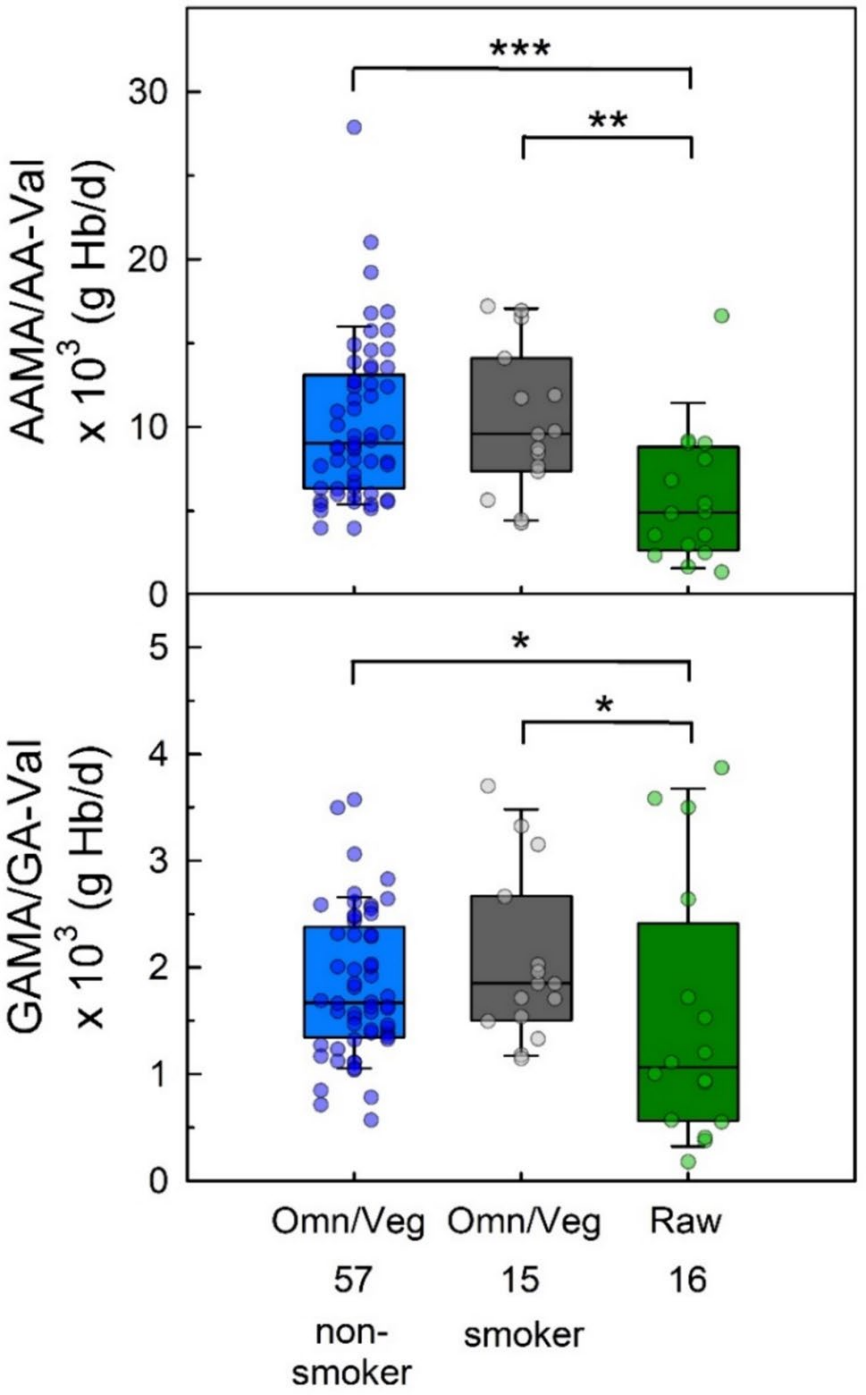
Ratios of daily AAMA excretion and AA-Val levels (in g Hb/day; upper panel) and of the daily GAMA excretion and GA-Val (lower panel) of 57 non-smoking omnivores and vegans (blue boxes), of 15 smoking omnivores and vegans (grey boxes), and of 16 raw food eaters (green boxes). Lines and boxes represent median values and the lower and upper quartiles, respectively, and the error bars represent the 10th and 90th percentiles. The significant differences are noted with asterisks (*p ≤ 0.05, **p ≤ 0.01, ***p ≤ 0.001, Mann–Whitney rank-sum test)

Comparing the excreted amounts of MAs of non-smoking omnivores with those of raw food eaters, the medians of the latter were found to be about 25% (AAMA) and 29% (GAMA) of those of omnivores (Table 1). In contrast, the medians of Hb adducts in raw food eaters were about 48% (AA-Val) and 43% (GA-Val) of those of non-smoking omnivores (Table 2). To shed more light on this matter, we calculated ratios (based on molecular units) between the two types of biomarkers (AAMA/AA-Val and GAMA/GA-Val) for non-smoking RBVD participants as well as for raw food eaters (Fig. 5; the ratios were alike for omnivores and vegans, wherefore the respective data were considered together in the following). The median ratio of AAMA/AA-Val in raw food eaters (4923 times more molecules excreted per day than measurable as adduct in one g Hb, in short: “g Hb/day﻿”) was about 54% of that observed in non-smoking omnivores/vegans (9037 g Hb/day). The difference was highly significant (p < 0.001). The median ratio of GAMA/GA-Val in raw food eaters (1068 g Hb/day) was about 64% of that observed in non-smoking omnivores/vegans (1669 g Hb/day, difference: p = 0.043).

The ratio of AAMA to GAMA was not found to be different between the groups of non-smoking vegans (median 10.7) and omnivores (median 10.6) as well as raw food eaters (median 11.5). In general, ratios containing GAMA of raw food eaters may be distorted by the number of seven urine samples with non-detectable GAMA, in which the concentration was replaced by LOD/2. The ratio of AA-Val to GA-Val was not different between nonsmoking vegans (median 2.18) and raw food eaters (median 2.27), but lower in non-smoking omnivores (median 1.87).

With respect to smokers in the group of omnivores/vegans, the ratios of AAMA/AA-Val (median 9596 g Hb/day) and GAMA/GA-Val (median 1852 g Hb/day) were not different from those in RBVD non-smokers (Fig. 5). The differences between smoking omnivores/vegans and raw food eaters were statistically significant for AAMA/AA-Val (p = 0.004) and for GAMA/GA-Val (p = 0.037).

### Urinary AAMA and GAMA excretion and AA-Val and GA-Val in blood samples of omnivores and vegans in 2017 and 2021

**Fig. 6:**
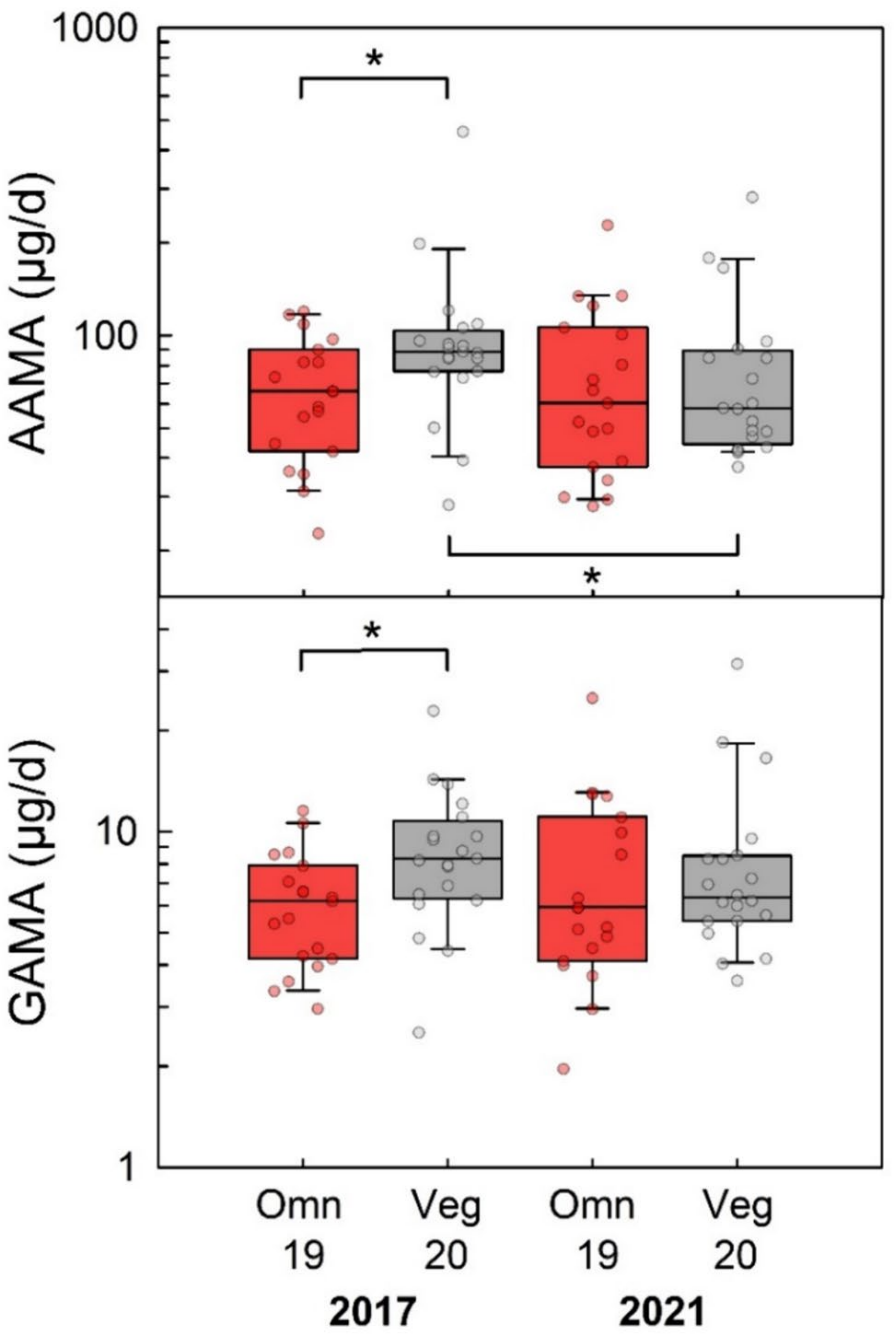
Daily excretion of AAMA (upper panel) and GAMA (lower panel) in non-smoking omnivore (red boxes, *n* = 19) and vegan (grey boxes, *n* = 20) study participants in 2017 (left columns) and in 2021 (right columns). Lines and boxes represent median values and the lower and upper quartiles, respectively, and the error bars represent the 10th and 90th percentiles. The significance of excretion differences is noted with an asterisk (*p ≤ 0.05, Mann–Whitney rank-sum test for the difference between vegans and omnivores and Wilcoxon signed-rank test for the pairwise difference between the data determined in 2017 and 2021)

The RBVD follow-up study allowed comparing the AAMA and GAMA excretion of non-smoking omnivores (n = 19) and vegans (n = 20) presenting themselves at the first and the second examination (Fig. 6). Smokers were not considered for a comparison. At the first investigation, the median daily AAMA excretions of omnivores and vegans were 66.0 (IQR 42.1, 90.2) µg/day and 88.5 (IQR 76.6, 104) µg/day, respectively (Fig. 6, left columns). In 2021, the median AAMA excretions of omnivores and vegans were 60.5 (IQR 37.5, 106) µg/day and 58.1 (IQR 44.4, 89.4) µg/day, respectively (Fig. 6, right columns). The time trend was not significant for omnivores (p = 0.71), but significant for vegans (p = 0.030). In the year 2017, the daily excreted GAMA amounts of omnivores and vegans were 6.2 (IQR 4.2, 7.9) µg/day and 8.2 (IQR 6.3, 10.7) µg/day, respectively. Four years later, the amounts of excreted GAMA of omnivores and vegans were 5.9 (IQR 4.1, 11.1) µg/day and 6.4 (IQR 5.4, 8.5) µg/day, respectively. The time trends were not statistically significant for omnivores (p = 0.29) and vegans (p = 0.15).

**Fig. 7:**
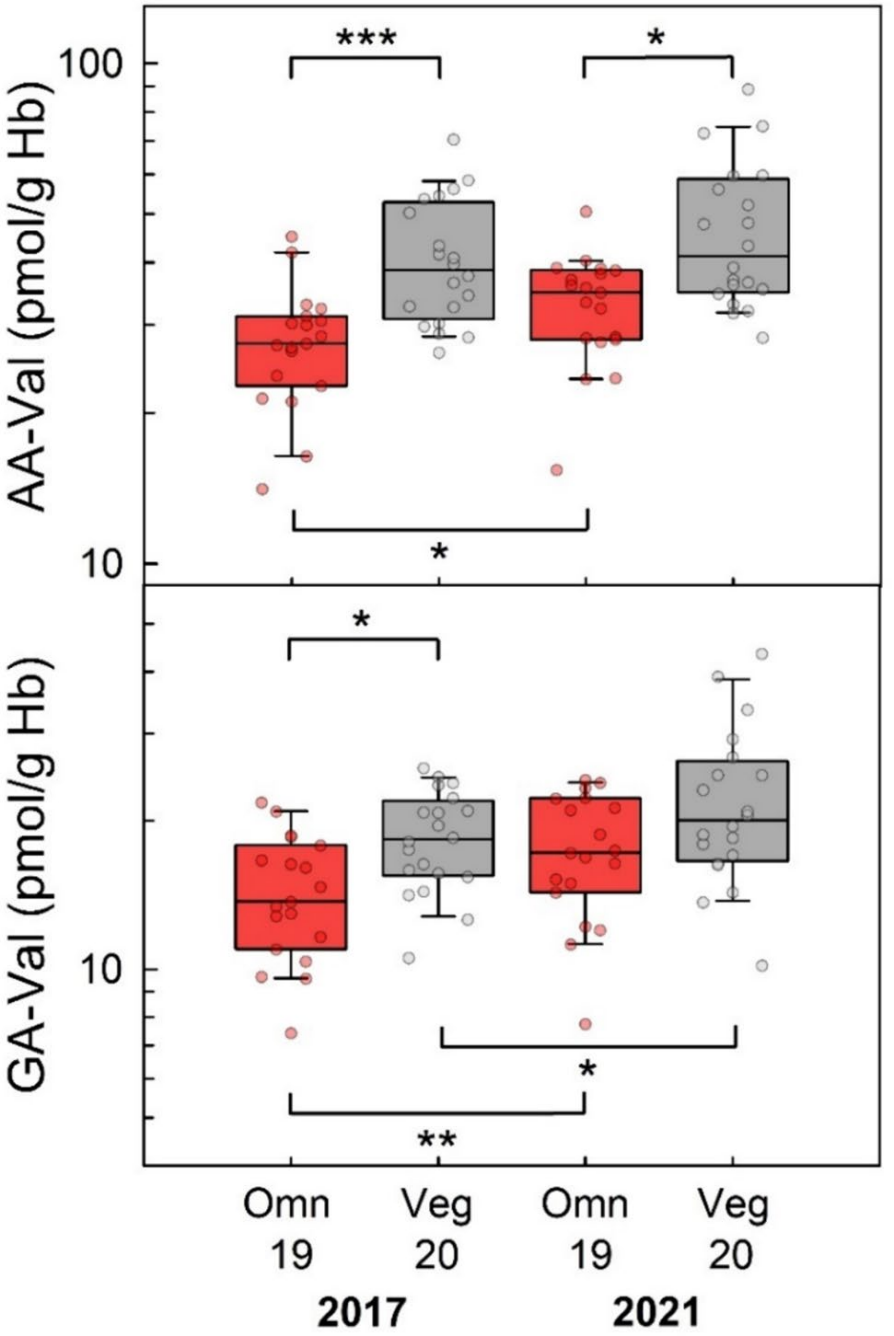
Levels of AA-Val (upper panel) and GA-Val (lower panel) in non-smoking omnivore (red boxes, *n* = 19) and vegan (grey boxes, *n* = 20) study participants in 2017 (left columns) and in 2021 (right columns). Lines and boxes represent median values and the lower and upper quartiles, respectively, and the error bars represent the 10th and 90th percentiles. The significance of adduct level differences is noted with asterisks (*p ≤ 0.05, **p ≤ 0.01, ***p ≤ 0.001, Mann–Whitney rank-sum test for the difference between vegans and omnivores and Wilcoxon signed-rank test for the pairwise difference between the data determined in 2017 and 2021)

The data on AA-Val and GA-Val in blood samples of non-smoking omnivores and vegans that were examined twice are summarized in Fig. 7. In 2017, median AA-Val levels in omnivores and vegans were 27.6 (IQR 22.7, 31.2) pmol/g Hb and 38.7 (IQR 30.8, 52.8) pmol/g Hb, respectively. In 2021, the median AA-Val levels in the same omnivore and vegan participants were 34.8 (IQR 28.1, 38.6) pmol/g Hb and 41.2 (IQR 34.9, 58.8) pmol/g Hb, respectively. The difference of AA-Val levels between both time points was statistically significant for omnivores (p = 0.012) but not for vegans (p = 0.058). The median GA-Val levels at the first examination were 13.7 (IQR 11.0, 17.8) pmol/g Hb in omnivores and 18.3 (IQR 15.5, 21.9) pmol/g Hb in vegans (Fig. 7). Four years later, the median GA-Val levels in omnivores and vegans were 17.2 (IQR 14.3, 22.2) pmol/g Hb and 20.0 (IQR 16.6, 26.4) pmol/g Hb, respectively. The time trends of GA-Val levels were significant for omnivores (p = 0.008) and vegans (p = 0.014).

### Correlations between the two measurements (2017 and 2021) of urinary MA excretion as well as of adduct levels

**Fig. 8:**
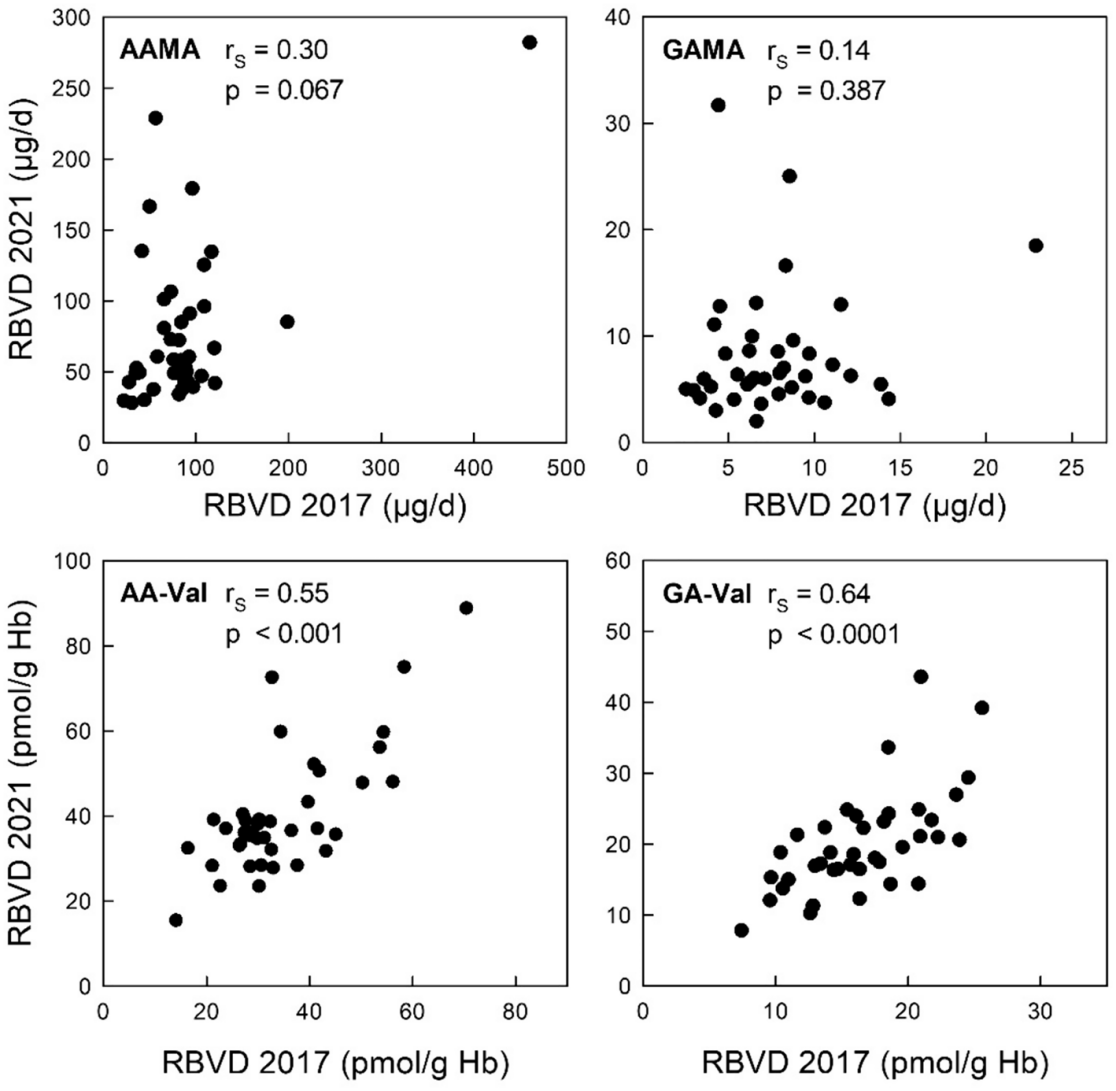
Scatterplots for the biomarkers determined in samples from 2017 (abscissae) and from 2021 (ordinates) in non-smoking vegans (*n* = 20) and omnivores (*n* = 19) of the RBVD study: urinary excretion of AAMA (upper left), GAMA (upper right); Hb adduct levels AA-Val (lower left) and GA-Val (lower right)

Figure 8 shows scatterplots of data on the daily excretion of AAMA and GAMA, and of the Hb levels of AA-Val and GA-Val determined in non-smoking participants of the RBVD study examined in 2017 and in 2021 (19 omnivores, 20 vegans). The weak correlations between the excreted amounts of MAs at both time points are not significant (AAMA: r_S_ = 0.30, p = 0.067; GAMA: r_S_ = 0.14, p = 0.39). In contrast, there is a positive correlation between levels measured four years apart for AA-Val (r_S_ = 0.55, p < 0.001) and for GA-Val (r_S_ = 0.64, p < 0.001). These differences are also reflected in the intraclass correlation coefficients (ICC) as a measure for stability of individual levels over time. The ICC was 0.11 for AAMA and 0.13 for GAMA, but 0.72 for AA-Val and 0.58 for GA-Val. According to Koo and Li (Koo and Li 2016), an ICC below 0.50 can be rated as low, and between 0.50 and 0.75 as moderate.

## Discussion

The focus of this article is on differences in short-term (MAs) and medium-term (Hb adducts) biomarkers of acrylamide resulting from different nutritional habits (omnivore, vegan, raw food). Due to the strict avoidance of the consumption of any warmed/heated food for at least four months (Abraham et al. 2022), the raw food eater study allows the monitoring of internal exposure to heat-induced contaminants with respect to their endogenous formation. The suitability of the study design to answer the question raised is, for example, demonstrated by the result that the urinary levels of 3-monochloro-1,2-propanediol (3-MCPD), a contaminant in processed oils and fats (Abraham et al. 2021), were under the limit of quantification in all 16 raw food eaters (article in preparation).

The RDVD study also included smoking omnivores and vegans. Their results confirm the well-known higher exposure via cigarette smoke, which were considered only for some special questions.

### Difference in internal acrylamide exposure between vegans and omnivores

Measurements of the arylamide biomarker—the Hb adducts AA-Val and GA-Val as well as the urinary MAs AAMA and GAMA—largely confirmed the levels and ratios measured by other groups of the general population. With respect to comparison of omnivores and vegans, Goerke et al. (2019) studied the exposure comparing the intake difference in small groups of non-smoking vegans and omnivores (n = 10 with 5 females each) in a duplicate study, analyzing the acrylamide content of all foodstuffs consumed over 10 days and the daily urinary excretion of AAMA and GAMA. Vegan participants ingested more acrylamide (mean 25.2 µg/day) compared to omnivores (mean 17.1 µg/day), and had a higher mean AAMA and GAMA excretion (87.6 µg/day and 12.5 µg/day, respectively) compared to omnivores (58.8 µg/day and 10.0 µg/day, respectively). These results were confirmed by our data revealing that median excretions of AAMA and GAMA in 24-h urine of the non-smoking vegans were higher than the corresponding values in the non-smoking omnivores. Likewise, our data on levels of the Hb adducts AA-Val and GA-Val levels (43% and 30% higher in vegans compared omnivores, respectively) confirmed a higher exposure to acrylamide from food consumption in vegans.

In the duplicate study by Goerke et al. (2019), the consumption of pan-fried vegetables, meat surrogates like tofu or seitan as well as bread-based products appeared to substantially contribute to the higher intake of acrylamide in vegans. In this context, results of the German food monitoring program are interesting, revealing significant levels of acrylamide in rice waffles, hash browns, potato pancakes, and vegetable chips (BVL 2021). Furthermore, the BfR MEAL Study (“meals for exposure assessment and analysis of food”, the first German Total Diet Study) very recently published the data on acrylamide, with highest levels in vegetable crisps (1430 µg/kg), followed by potato pancakes (558 μg/kg) and pan-fried potatoes (450 μg/kg) (Perestrelo et al. 2024). As vegans have to safeguard their energy requirements with a reduced range of meals, a high consumption of these food groups in general is a plausible explanation for the higher internal exposure to acrylamide in vegans compared to omnivores.

### Urinary excretion of MAs of acrylamide in raw food eaters

Based on the urinary excretion data observed in rats (levels of AAMA and GAMA in untreated animals similar to those of ^14^C-AAMA and ^14^C-GAMA after application of a single oral dose of 0.1 µg ^14^C-acrylamide/kg bw (Watzek et al. 2012)) and in humans after a washout phase of three to five days with an acrylamide-minimized diet (Goempel et al. 2017; Ruenz et al. 2016), an endogenous formation of acrylamide was hypothesized. In the latter studies, a significant mean urinary AAMA excretion between 0.09 and 0.14 µmol/day was observed after the washout phases. This is somewhat higher compared to the median AAMA excretion of 0.066 µmol/day (15.4 µg/day) in the raw food eaters. The difference may be due to the fact that the acrylamide-minimized diets do not reduce the amount of acrylamide in food that consequently compared to food not heated to higher temperatures than 42 °C. Compared to the non-smoking RBVD omnivores, the median AAMA excretion of the raw food eaters was about 25% (Table 1). This is comparable to the values of 32% and 27% calculated from the mean washout data of Goempel et al. (2017) for group A (washout 9 days) and group B (washout 13 days), respectively, in comparison to the mean AAMA excretions of the respective groups on day 1 (n = 6 omnivores each). These aggregated results would suggest that roughly one-fourth to one-third of the AAMA excretion in omnivores may occur independently of the dietary acrylamide intake.

As an attempt to express this endogenous acrylamide dose as a corresponding daily external dose, reverse dosimetry was applied in the past, e.g., using the data of Goempel et al. (2017) who determined a fraction of urinary ^3^C_3_D_3_-AAMA excretion of 41% (on a molecular base) four days after oral application of an aqueous solution with a dose of 1 µg ^13^C_3_D_3_-acrylamide in six adults per kg bw. As a result, estimations of the corresponding daily external doses between 0.2 and 0.4 µg/kg bw have been published (Goempel et al. 2017; Ruenz et al. 2016). However, these calculations not only assume a bioavailability of 100% [as realistic for aqueous solutions, but may be lower in case of solid foods Berger et al. 2011; Doerge et al. 2005b)], but also a metabolic fate after oral administration comparable to that of endogenous acrylamide (see discussion below).

### Hb adducts of acrylamide in raw food eaters

Due to the strict avoidance of the consumption of any warmed/heated foods in the raw food eaters for at least four months, our study allowed to answer questions of possible endogenous formation of heat-induced contaminants not only using short-term biomarkers like mercapturic acids, but also using Hb adducts as more stable medium-term biomarkers. As mentioned above, the median AAMA excretion of the raw food eaters was about 25% compared that of non-smoking RBVD omnivores. In contrast, a ratio of 48% was calculated from the median levels of Hb AA-Val in raw food eaters and those of non-smoking RBVD omnivores. These data of the raw food eaters are the only available, no other data on AA-Val in people strictly avoiding dietary acrylamide exposure for at least four months (the life time of Hb adducts) have been published. Our data indicate an estimated systemic acrylamide exposure in raw food eaters nearly half as high as that of non-smoking omnivores. The difference in biomarker results for urinary AAMA excretion (25%) and for AA-Val (48%) is also reflected in the ratios of AAMA and AA-Val as well as of GAMA and GA-Val (Fig. 5), showing significantly higher ratios in the RBVD participants compared to the raw food eaters.

At first glance, this seems surprising, but may indicate a different metabolic fate of dietary (oral) acrylamide and endogenous acrylamide. Notably, after oral exposure and intestinal absorption, acrylamide undergoes hepatic first-pass metabolism, leading to significant turnover of acrylamide to glycidamide and to detoxification of acrylamide through glutathione conjugation and other pathways. This has been demonstrated in rodents (Doerge et al. 2005a, b), resulting in different internal exposure of acrylamide and glycidamide upon oral uptake in comparison to intravenous administration. The latter reflects the situation in case of an endogenous source of acrylamide. Humans are less competent to metabolize acrylamide to glycidamide than rodents, but detoxifying biotransformations, especially coupling of acrylamide and glycidamide to glutathione, are more efficient in humans than in rodents (Berger et al. 2011; Fennell and Friedman 2005; Fuhr et al. 2006; Rietjens et al. 2022). In view of our data, a relevant first-pass metabolism with relatively high glutathione conjugation is plausible and would explain the differences between the RBVD participants (vegan or omnivore nutrition) and the raw food eaters observed in our studies. Thus, a higher proportion of AAMA (and GAMA) was formed from acrylamide after oral exposure compared to acrylamide from endogenous sources. Therefore, the use of a fraction of urinary AAMA excretion determined after oral acrylamide administration (e.g., 41% on a molecular base by Goempel et al. (2017), see above) for reverse dosimetry may provide an underestimation of the corresponding external dose of acrylamide, if this re-calculation is done with urinary AAMA data from raw food eaters or people with minimized acrylamide intake for several days. In these cases, a fraction of urinary AAMA excretion determined following an intravenous application would be more appropriate, but is not available.

A generally accepted model for reverse dosimetry using the AA-Val values (as medium-term biomarkers reflecting the systemic exposure) is not available, but an estimation is possible using data of the study in humans by Fennel et al*.* (2005). They applied single oral doses (0.5, 1.0, or 3.0 mg/kg bw in aqueous solutions) of ^13^C-acrylamide to male participants (n = 5 per group), and measured ^13^C-AA-Val and ^13^C-GA-Val levels linearly increasing with the dose. On average, one mmol ^13^C-acrylamide per kg bw led to mean ^13^C-AA-Val levels of 74.7 nmol per g globin. Using this value and considerations for reverse dosimetry^2^ (Fennell et al. 1992; Hays and Aylward 2008), an external dose of 0.22 µg/kg bw was calculated from the median AA-Val level in the raw food eaters of 13.3 pmol/g Hb. A dose of acrylamide in this range representing endogenous exposure would add to the external dose of dietary acrylamide, which was estimated to be 0.4 µg/kg bw per day on average in German adults of the general population (EFSA 2015).

Finally, a small note in connection with the hepatic first-pass effect: It may be confusing that smokers of the RBVD study have about the same ratios of AAMA/AA-Val as the non-smokers (Fig. 5), as the former have a relevant acrylamide exposure from inhalation of cigarette smoke not undergoing a hepatic first-pass effect. However, data in rats (Doerge et al. 2005b; Sumner et al. 2003) may indicate a pulmonary first-pass metabolism after inhalation similar to the hepatic first-pass metabolism after oral exposure (IRIS 2010).

### Possible sources of internal acrylamide exposure in raw food eaters

Throughout this article, the term “endogenous” was used to describe the source of the internal exposure to acrylamide in raw food eaters, knowing that this is a simplification. Firstly, an exposure via the dermal route (e.g., by hair and skin care products (Kraeling and Bronaugh 2005)) may theoretically be possible, but evidence for a relevant exposure in the general population is missing. Furthermore, dermal bioavailability was found to be low (Fennell et al. 2005). Secondly, inhalational exposure from second-hand tobacco smoke and smoke from barbecuing and campfires (Goerke et al. 2019) is possible, but all raw food eaters were asked and denied to be exposed to this kind of smoke. Thirdly, the foodstuffs consumed by raw food eaters may contain acrylamide, even if not heated at temperatures > 120 °C. Dried fruits are known to possibly contain acrylamide without such a heat-treatment. Surma et al. (2018) reported acrylamide levels in dates (3 out of 4 brands; 20.1 to 50.9 µg/kg), in apricots (5 out of 6 brands; 13.5 to 100 µg/kg), and in plums (6 out of 6 brands; 22.1 to 141 µg/kg). The mechanism(s) of acrylamide formation during drying are not known, although two of the required conditions are met: the fruits mentioned contain high levels of the precursor asparagine (Amrein et al. 2007; Bahrami et al. 2021; Lo Voi et al. 1995), and the decreasing moisture content favors the Maillard reaction and the formation of acrylamide (Lund and Ray 2017). Amrein et al. (2007) commented that the drying process may take several days entailing temperatures reaching 70 to 80 °C, which is probably not known to all raw food eaters. Using the three-day weighed food records, the consumption of dried fruits was documented and revealed no consumption in 7 participants, a moderate consumption in 8 participants (average 70 g/day, range 10 to 179 g/day), and an extreme consumption (797 g/day) in one participant. The latter was the one with an extreme urinary AAMA excretion (but unremarkable Hb AA-Val levels), and a later analysis of “Medjool” dried dates revealed a high level of acrylamide (see results chapter; urinary AAMA excretion of this participant was replaced the results by a new 24-h urine collected on a day without prior consumption of dates). Urinary AAMA excretions of raw food eaters having consumed dried fruits during the 3-day food recordings were not significantly different from those of the non-consumers. Therefore, we would exclude a relevant contribution of acrylamide from dietary exposure in our group of raw food eaters, and consider their internal exposure to acrylamide as a result of endogenous formation.

The causes of such an endogenous formation of acrylamide in the organism is under scientific discussion. Oxidative stress is one of the hypotheses. Tareke et al. (2008) proved this hypothesis by feeding mice with substances known to induce formation of reactive oxygen species, and observed an increase of AA-Val and GA-Val levels. The picture was completed by demonstrating that acrylamide is formed at physiological conditions (37 °C, pH 7.4), when asparagine is incubated in the presence of hydrogen peroxide (Tareke et al. 2009). Rietjens et al. (2022) hypothesized that the addition of ammonia to acrolein may lead to the formation of acrylamide, and generation in the gut may also play a role. In humans, a positive association between the urinary excretions of AAMA and the biomarker for oxidative stress, 8-hydroxy-2´-deoxyguanosine, was observed in adolescents and young adults (n = 800) (Lin et al. 2013). According to the literature evaluation of EFSA (EFSA 2015), various parameters, *i.e.* BMI, alcohol consumption, sex, age or metabolic genotype, may have an impact on the urinary excretion of AAMA and GAMA and the formation of AA-Val and GA-Val levels in Hb.

### Time trend of acrylamide exposure between 2017 and 2021 in the RBVD study

Since the discovery of relevant dietary expose to acrylamide in 2002, mitigation strategies (current regulation in the European Union: 2017/2158) led to reduction of acrylamide levels in some food classes like potato crisps, but for other food categories, no stable trends of acrylamide were observed in Europe (EFSA 2015; Mojska and Gielecińska 2024). With respect to the biomarker levels of our study, we observed significant increases of AA-Val and GA-Val in non-smoking omnivores (26% each) and in vegans (6.5% and 9.3%, respectively). These results do not support that mitigation strategies in recent years are successful. Data on urinary AAMA and GAMA excretion showed a slight decrease in vegans only. However, far-reaching conclusions should be avoided as the numbers of participants were relatively low.

### Validity of AAMA/GAMA and AA-Val/GA-Val as biomarkers of individual exposure

The interpretation of our data goes hand in hand with a critical consideration about the validity and scope of the biomarkers. Daily excreted amounts of MAs are short-term biomarkers of exposure to the parent compounds; however, the individual short-term variability of exposure usually is unknown and depends on the relative constancy of the dietary habits. In this regard, Hb adducts provide a higher reliability because they represent an integrated mean level of plasma concentrations over a few months, and thus are indifferent to daily extremes of exposure (Neumann 1984; Wild 2009). Using the RBVD data of 39 participants from 2017 and 2021, we evaluated the intra-individual stability over time, a particular requirement in order to study associations between exposure and slowly evolving adverse health effects like cancer (Barregard et al. 2013; Jungert and Frank 2021). As expected, correlations of individual levels (Fig. 8) were found to be weak for urinary AAMA and GAMA excretion. These results as well as the corresponding ICCs underline that a single measurement of the daily excretion of the MAs does not adequately reflect the individual exposure at a certain time. The corresponding results of the pairs of AA-Val and GA-Val levels determined four years apart demonstrated much better correlations and higher ICCs, confirming the significance of Hb adducts as biomarkers of exposure.

In this context, the correlations between the excreted amounts of AAMA and GAMA and the respective medium-term biomarkers AA-Val and GA-Val observed in our study in non-smoking participants of the RBVD study (Fig. S2 in the Supplemental Information, data of 2017) were moderate only (r_S_ = 0.50 and 0.58, respectively), emphasizing a high day-to-day variability and the limited capability of the MAs to replace Hb adducts as biomarkers of exposure. However, in epidemiological studies on acrylamide exposure (e.g. Poteser et al. (2022)), often only urinary AAMA and GAMA are measured in spot urine (which is available more easily than 24-h urine), and the results often are given as concentration (µg/L), but were not based on urinary creatinine levels to adjust for the hydration status [which, however, may be misleading for the comparison of vegans and omnivores (Abraham et al. 2023)]. Such data may only be applicable to compare acrylamide exposure in different populations. Compared to AAMA and GAMA, the Hb adducts AA-Val and GA-Val reflect the systemic exposure and are superior biomarkers due to their time stability and significance. For better reverse dosimetry, a controlled exposure study in humans would be needed to establish a reliable adduct level increase per dose factor, allowing the estimation the average external acrylamide exposure from AA-Val, as shown previously for glycidol and its Hb adduct *N*-(2,3-dihydroxypropyl)-Val (Abraham et al. 2019).

## Conclusions

Our data increase the evidence of a significant endogenous contribution to the overall internal exposure to acrylamide, which is unrelated to the external exposure. Results of the strict raw food eaters—abstaining from any warmed or heated food for at least four months—for the first time allowed to base the detection of endogenous acrylamide on Hb adducts. These data revealed a relatively high endogenous formation, reaching in case of AA-Val nearly half the value of omnivores. Basing the comparison on urinary AAMA excretion, endogenous formation would be much lower. This discrepancy likely is due to missing hepatic first-pass metabolism of acrylamide in case of the raw food eaters, leading to a lower extent of glutathione conjugation and to a higher systemic exposure.

One open question remains: how to consider the additional endogenous exposure in risk assessment? With respect to toxicological data on effects in laboratory animals, their endogenous exposure can be expected to be negligible, as in such settings with few animals used per dose group, measurable effects are expected at doses much higher than the dose corresponding to an endogenous formation of acrylamide. With respect to the exposure estimation in humans using consumption and occurrence data, the endogenous exposure has to be added as long as no data are available on differences in biological response between exogenous and endogenous acrylamide. More data on endogenous sources of arylamide is needed to better understand the internal exposure and its contribution to the overall risk.

### Supplementary Information

Below is the link to the electronic supplementary material.

Supplementary file1 (PDF 943 KB)

## Data Availability

The datasets generated during and/or analysed during the current study are not publicly available due to a lack of consent from study participants but are available from the corresponding author on reasonable request.

## Abbreviations

AAMA: Acrylamide mercapturic acid (*N*-acetyl-S-(2-carbamoylethyl)-L-cysteine)
AA-Val: *N*-(2-Carbamoylethyl)-Val
bw: Body weight
EFSA: European Food Safety Authority
FITC: Fluorescein-5-isothiocyanate
FTH: Fluorescein thiohydantoin
Hb: Hemoglobin
GAMA: Glycidamide mercapturic acid (*N*-acetyl-*S*-(2-carbamoyl-2-hydroxyethyl)-L-cysteine)
GA-Val: *N*-(2-Carbamoyl-2-hydroxyethyl)-Val
IPC: Ion-pair liquid chromatography
IPC-MS/MS: Ion-pair liquid chromatography-tandem mass spectrometry
LC–MS/MS: Liquid chromatography-tandem mass spectrometry
LOD: Limit of detection
LOQ: Limit of quantification
MA: Mercapturic acid
MRM: Multiple reaction monitoring
RBVD study: Risks and Benefits of a Vegan Diet Study
S/N: Signal-to-noise ratio
SPE: Solid-phase extraction
UPLC-MS/MS: Ultra-performance liquid chromatography-tandem mass spectrometry

## Footnotes

1 Landesbetrieb Hessisches Landeslabor (LHL).

2 As conceptually described by Fennel et al. (1992), the expected amount of hemoglobin adducts accumulating in a chronic steady-state exposure can be calculated using data of the adduct amount formed per day and the kinetics of RBC turnover (lifespan of red blood cells: 120 days, *i.e.* the adduct levels formed after an acute dose would be present in blood for 60 days on average). The small difference between globin- and hemoglobin-based adduct levels (factor 1.04) was considered.
