# Supplementary material for: Internal exposure to heat-induced food contaminants in omnivores, vegans and strict raw food eaters: biomarkers of exposure to acrylamide (hemoglobin adducts, urinary mercapturic acids) and new insights on its endogenous formation
Source: Arch Toxicol. 2024 May 31;98(9):2889–905. doi: 10.1007/s00204-024-03798-z (PMC11324683; doi:10.1007/s00204-024-03798-z)
Supplement: Supplementary file 1 — Supplementary file1 (PDF 943 KB) [file 204_2024_3798_MOESM1_ESM.pdf]

Internal Exposure to Heat-induced Food Contaminants in Omnivores, Vegans and Strict Raw Food Eaters: Biomarkers of Exposure to Acrylamide (Hemoglobin Adducts, Urinary Mercapturic Acids) and New Insights on its Endogenous Formation

Bernhard H. Monien, Nick Bergau, Fabian Gauch, Cornelia Weikert, Klaus Abraham

Supplemental Information

Validation of LC–MS/MS quantification of AAMA and GAMA

AAMA and GAMA concentrations in urine samples were determined by isotope-dilution LC-MS/MS. The preceding dilute-and-shoot approach used here was simpler in comparison to earlier works, in which solid-phase extraction (SPE) with reversed-phase cartridges was applied (Eckert et al. 2010; Jia et al. 2019; Ruenz et al. 2016). The sensitivities of mass spectrometric detection, and the abundance of AAMA and GAMA in urine samples of study participants with omnivore and vegan diets allowed detection and quantification even after a 1:9 dilution with water, which greatly enhanced time and cost effectiveness in comparison to the SPE method. The efficiency of ion-pair reversed-phase chromatography, previously proven for the separation of other polar carboxylic acids in urine samples (Bergau et al. 2021), also added to the sensitivity of detection.

The linear range of the quantification methods as well as the limits of detection (LOD) and the limits of quantification (LOQ) of the analytical method were determined with the deuterated standards, because of the inevitable presence of AAMA and GAMA in the urine sample used for the validation. Consequently, the resulting limit values are approximate values (Table S3). Dilution series with 16 concentrations between 1 ng/L and 100 µg/L of d_3-AAMA and d_3-GAMA were prepared in water and with equivalent concentrations also in the presence of urinary matrix from a urine pool of 5 subjects. The LOD and LOQ values of the analyses were defined by signal-to-noise ratios (S/N) of 3 (LOD) and 10 (LOQ). The LOD values observed in the presence of urinary matrix for d_3-AAMA and d_3-GAMA (0.025 µg/L, 50 fg on column) reflect a high sensitivity. The application of urine volumes between 50 µL and 4 mL in 18 previous analytical studies led to LOD values of 0.05 – 80 µg/L for AAMA and 0.05 – 10 µg/L for GAMA (Albiach-Delgado et al. 2022). The viable results of our method and five of the 18 studies employing a dilute-and-shoot approach included in the overview of Albiach-Delgado *et al.* (LOD values for AAMA 0.1 – 80 µg/L) support our notion that the AAMA analysis does not benefit from SPE enrichment, probably because of the multitude of co-eluting urinary matrix interfering with mass spectrometric detection. The linear regression analyses of the mass spectrometric responses yield coefficients of determination (R^2) of > 0.999 (Fig. S3). The ratios of the calibration line slopes determined from workup of aqueous solutions or in the presence of pooled urine reflect the minor influence of the urinary matrix after ten-fold dilution of the urine samples. A small positive matrix-effect was observed for d_3-AAMA with a slight signal increase of about 6% in the presence of urinary matrix. In contrast, the signal intensities of d_3-GAMA decreased by about 23% if urine was present in the sample.

The inter- and intraday precision data were determined by spiking of d_3-AAMA and d_3-GAMA to urine pool samples at three concentrations corresponding to 2×LOQ, 10×LOQ and

50×LOQ (Table S3). The values meet the criteria for the inter- and intraday precision of biomarker analyses by chromatographic assays (not to exceed ± 15%, except ± 20% at the LOQ) stated by the Food and Drug Administration (U. S. Department of Health Human Services 2018).

Occurrence and handling of signals below the limit of quantification

Due to the varying background noise in all urine samples, the S/N of individual AAMA and GAMA signals was calculated separately for the evaluation of their validity. The S/N values of the AAMA peaks observed in the 138 samples (RBVD study, n = 122; raw food eaters, n = 16) were between 14.2 and 8310. The GAMA excretion in the RBVD study was well detectable with S/N values between 4.5 and 224 and ten signals < LOQ. In urine samples of raw food eaters, seven signals were < LOD, seven signals were between the LOD and the LOQ, and two signals were > LOQ. For the evaluation, all GAMA signals with S/N between the LOD and the LOQ were used as such, because a higher validity of results can be expected compared to the replacement with half of the LOQ (EFSA Working Group on Left Censored Data 2010). The GAMA concentrations in urine samples of raw food eaters with non-detects were set to LOD/2. Representative chromatography runs for the quantification of AAMA and GAMA in a urine sample of one RBVD study participant are depicted in Fig. S4.

The method for the quantification of AA-Val and GA-Val in Hb using a FITC-mediated Edman degradation was validated previously (Gauch et al. 2022). In this study, all AA-Val signals (138 samples) were well above the LOQ with S/N = 49.6 as the lowest value. In case of GA-Val, one sample signal of the RBVD study and four of the raw food eater study were between the LOD and the LOQ. As recommended, the GA-Val levels were used as such for the evaluation (EFSA Working Group on Left Censored Data 2010).

Table S1. Parameters for the mass spectrometric detection of the FTH conjugates resulting from the FITC-mediated cleavage of modified Val residues from Hb and of the respective isotope-labeled standard compounds

analyte	RT min	transition	Q1 m/z	Q3 m/z	DP V	EP V	CE V	CXP V
AA-Val-FTH	12.34	quantifier	560.2	445.0	190	8	58	20
		qualifier 1		374.0	190	8	61	20
		qualifier 2		358.0	190	8	80	20
AA-d_7-Val-FTH	12.29	quantifier	567.2	445.0	190	8	58	20
		qualifier 1		374.0	190	8	61	20
		qualifier 2		358.0	190	8	80	20
GA-Val-FTH	11.70	quantifier	576.2	531.0	200	8	49	20
		qualifier		489.0	200	8	56	20
GA-d_7-Val-FTH	11.64	quantifier	583.2	538.0	200	8	49	20
		qualifier 1		489.0	200	8	56	20
		qualifier 2		496.1	200	8	48	20

Table S2. Mass spectrometric parameters for the detection of the urinary analytes AAMA and GAMA and the respective isotope-labeled standards d_3-AAMA and d_3-GAMA used for quantification.

analyte	RT min	transition	Q1 m/z	Q3 m/z	DP V	EP V	CE V	CXP V
AAMA	6.24	quantifier	233.0	104.0	-30	-8	-18	-15
		qualifier		162.0	-30	-8	-13	-15
d_3-AAMA	6.21	quantifier	236.0	104	-30	-8	-18	-15
GAMA	5.08	quantifier	249.0	120.0	-30	-8	-20	-15
		qualifier		128.0	-30	-8	-14	-15
d_3-GAMA	5.05	quantifier	252.0	120.0	-30	-8	-20	-15

Table S3. Validation parameters for the analyses of d_3-AAMA and d_3-GAMA in aqueous solution and in human urine samples.

			d_3-AAMA	d_3-GAMA
**in aqueous solution**				
**linear detection range**[a,b]		µg/L	0.025 - 100	0.025 - 100
**LOQ**[a,b]		µg/L	0.1	0.05
**with urinary matrix**				
**linear detection range**[a,b]		µg/L	0.025 - 100	0.05 - 100
**LOQ**[a,b]		µg/L	0.1	0.25
**intraday precision (CV)**[c]	low	%	15.9	15.7
	medium	%	8.4	12.3
	high	%	4.9	1.4
**interday precision (CV)**[c]	low	%	18.1	17.6
	medium	%	6.9	9.6
	high	%	5.9	4.4

a The validation was conducted using the isotope-labelled compounds, because human urine samples contain high background levels of AAMA and GAMA.

b The linearity of detection and the LOD (S/N = 3) and LOQ values (S/N = 10) were determined using 16 solutions of d_3-AAMA and d_3-GAMA (1 ng/L to 100 µg/L), which were either prepared in water or in the presence of biomatrix (Fig. S3). The LOD values are the lower numbers stated for the linearity range.

c The precision was determined using spiked urine samples with d_3-AAMA concentrations of 0.2 µg/L (low, 2×LOQ), 1.0 µg/L (medium, 10×LOQ) and 5.0 µg/L (high, 50×LOQ) and d_3-GAMA concentrations of 0.5 µg/L (low, 2×LOQ), 2.5 µg/L (medium, 10×LOQ) and 12.5 µg/L (high, 50×LOQ).

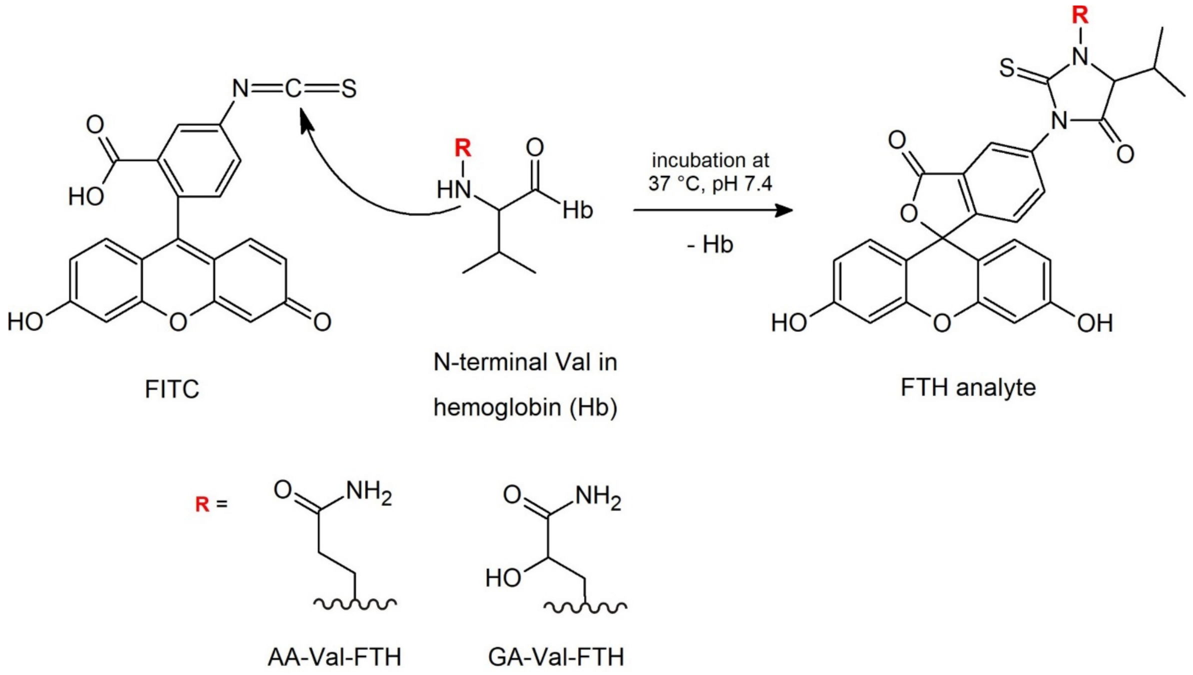

Fig. S1 The Edman degradation with FITC leads to formation of fluorescein thiohydantoin (FTH) conjugates. The FTH analyte is specific for the Val modified with a reactive electrophile (R).

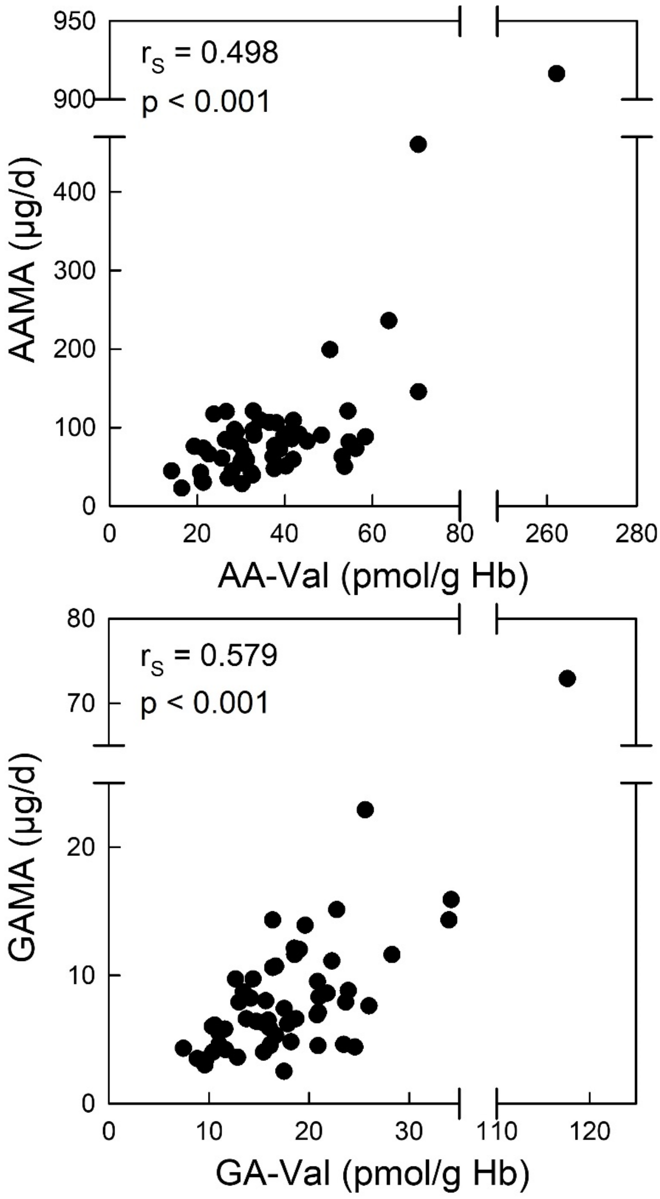

Fig. S2 Scatterplots of the urinary excretion of mercapturic acids (ordinates) and Hb adduct levels (abscissae) in non-smoking vegans and omnivores (n = 31/26) of the RBVD study (2017): daily AAMA excretion and AA-Val in Hb (upper panel), daily GAMA excretion and GA-Val in Hb (lower panel). Moderate significant correlations are observed between the daily AAMA excretion and AA-Val in Hb (r_S = 0.498, $p < 0.001$) and between the GAMA excretion and GA-Val levels (r_S = 0.579, $p < 0.001$).

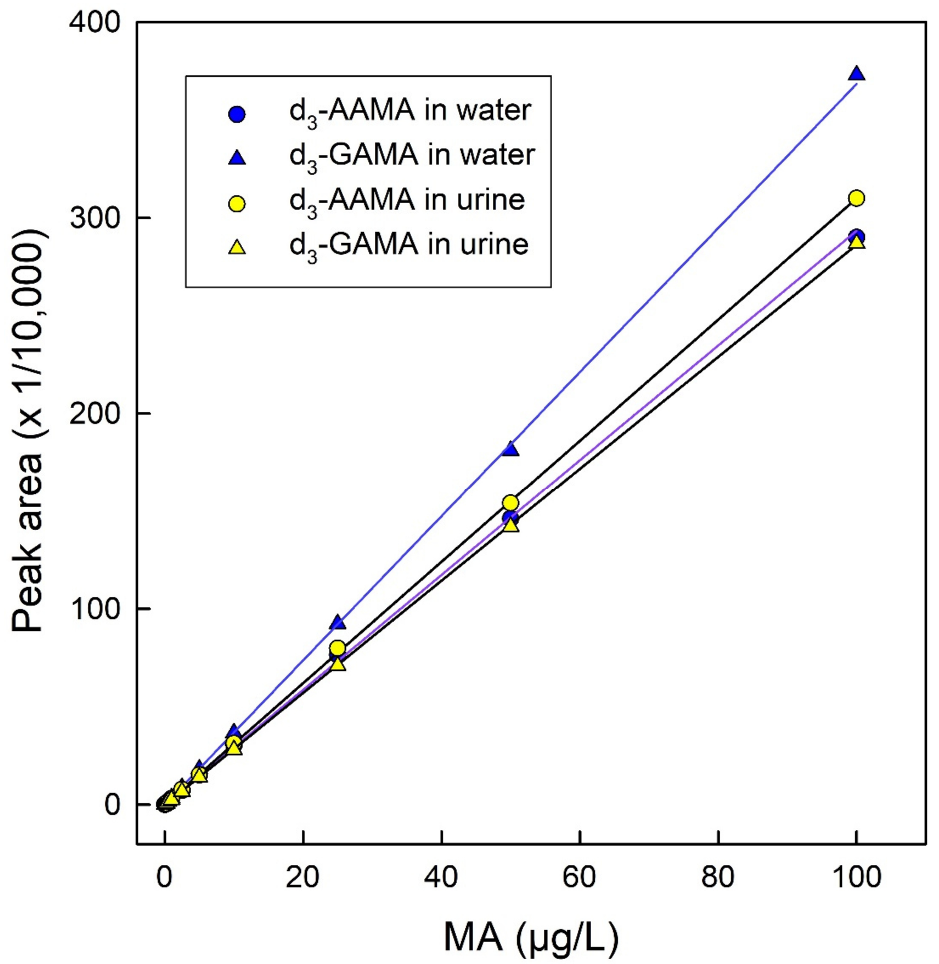

Fig. S3 Peak areas of d_3-AAMA detection (*m/z* = 236 → 104) and d_3-GAMA detection (*m/z* = 252 → 120) recorded after direct injection of 16 aqueous samples with concentrations in the range of 1 ng/L and 100 µg/L (blue dots). The data was fitted with a trend line by least-squares linear regression (R^2 > 0.999). Concentrations of AAMA and GAMA were also determined in the presence of processed ´blank´ urine samples (pooled urine of five individuals; yellow dots). The data was also fitted by linear regression (R^2 > 0.999). The comparison of the slopes allowed estimating matrix-dependent alterations of the mass spectrometric signal.

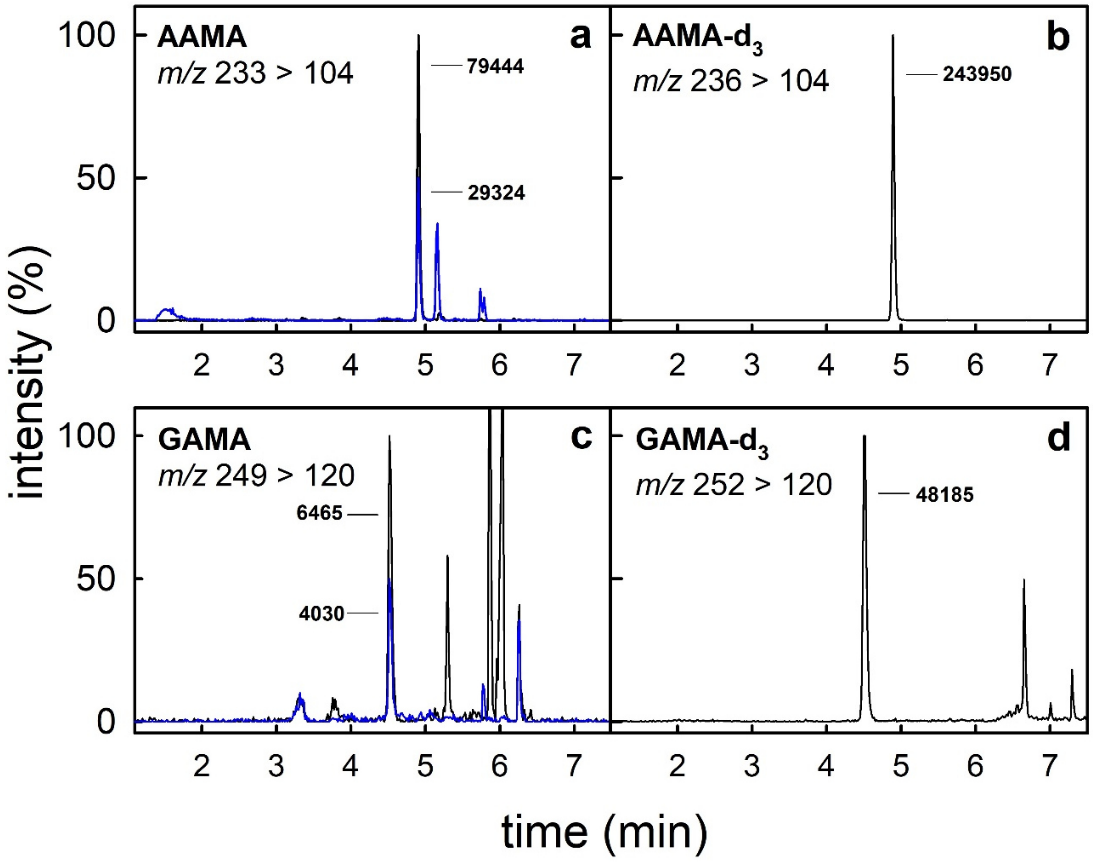

Fig. S4 LC-MS/MS chromatograms of the quantifier traces (black lines) from AAMA (*m/z* 233 → 104; panel a) and GAMA (*m/z* 249 → 120; panel c) after addition of the deuterated compounds and dilution of the urine sample with water. The peaks of the quantifier and the qualifier signals (blue lines) were normalized to 100% and 50% signal intensity, respectively. The absolute intensities (peak areas) of analyte peaks are given. The panels on the right hand side show the signals recorded for the deuterated compounds AAMA-d_3 (*m/z* 236 → 104; panel b) and GAMA-d_3 (*m/z* 252 → 120; panel d).

References

Albiach-Delgado A, Esteve-Turrillas FA, Fernandez SF, Garlito B, Pardo O (2022) Review of the state of the art of acrylamide human biomonitoring. Chemosphere 295:133880 https://doi.org/10.1016/j.chemosphere.2022.133880

Bergau N, Zhao Z, Abraham K, Monien BH (2021) Metabolites of 2- and 3-Monochloropropanediol (2- and 3-MCPD) in Humans: Urinary Excretion of 2-Chlorohydracrylic Acid and 3-Chlorolactic Acid after Controlled Exposure to a Single High Dose of Fatty Acid Esters of 2- and 3-MCPD. Mol Nutr Food Res 65:e2000736 https://doi.org/10.1002/mnfr.202000736

Eckert E, Drexler H, Goen T (2010) Determination of six hydroxyalkyl mercapturic acids in human urine using hydrophilic interaction liquid chromatography with tandem mass spectrometry (HILIC-ESI-MS/MS). J Chromatogr B 878:2506-2514 https://doi.org/10.1016/j.jchromb.2009.09.003

EFSA Working Group on Left Censored Data (2010) Management of left-censored data in dietary exposure assessment of chemical substances. EFSA J 8:96

Gauch F, Abraham K, Monien BH (2022) Simultaneous quantification of eight hemoglobin adducts of genotoxic substances by isotope-dilution UHPLC-MS/MS. Anal Bioanal Chem 414:5805-5815 https://doi.org/10.1007/s00216-022-04143-y

Jia W, Wu D, Chen X, et al. (2019) Metabolomics-based biomarker analysis of dihydroxypropyl mercapturic acid isomers from 3-monochloropropane-1,2-diol and glycidol for evaluation of toxicokinetics in rats and daily internal exposure in humans. Talanta 204:329-336 https://doi.org/10.1016/j.talanta.2019.06.009

Ruenz M, Bakuradze T, Eisenbrand G, Richling E (2016) Monitoring urinary mercapturic acids as biomarkers of human dietary exposure to acrylamide in combination with acrylamide uptake assessment based on duplicate diets. Arch Toxicol 90:873-881 https://doi.org/10.1007/s00204-015-1494-9

U. S. Department of Health Human Services (2018) Bioanalytical Method Validation, Guidance for Industry. Food and Drug Administration
